# Development and validation of a new co-dominant DNA marker for selecting the null allele of polyphenol oxidase gene *Ppo-D1* in common wheat (*Triticum aestivum* L.)

**DOI:** 10.1270/jsbbs.24071

**Published:** 2025-04-04

**Authors:** Akiko Nakamaru, Keita Kato, Sachiko Ikenaga, Toshiki Nakamura, Katsunori Hatakeyama

**Affiliations:** 1 Tohoku Agricultural Research Centre, National Agricultural and Food Research Organization, 4 Akahira, Shimo-kuriyagawa, Morioka, Iwate 020-0198, Japan; 2 The United Graduate School of Agricultural Sciences, Iwate University, 3-18-8 Ueda, Morioka, Iwate 020-8550, Japan; 3 Western Region Agricultural Research Centre, National Agricultural and Food Research Organization, 6-12-1 Nishifukatsu, Fukuyama, Hiroshima 721-8514, Japan; 4 Faculty of Agriculture, Iwate University, 3-18-8 Ueda, Morioka, Iwate 020-8550, Japan

**Keywords:** wheat, PPO, *Ppo-D1*, *Ae. tauschii*

## Abstract

Polyphenol oxidase (PPO) is a key enzyme contributing to the time-dependent discoloration of wheat products. Developing cultivars with low PPO activity is one way to solve this problem. In this study, we focused on the *Ppo-D1* gene, which has the second highest effect on grain PPO activity after the *Ppo-A1* gene. Utilizing resequencing data, we found that the *Ppo-D1* gene in the common wheat line ‘Fukuhonoka-NIL’, which exhibits low PPO activity, has an approximately 3 kb deletion in the 3′UTR and a 73 bp deletion in the third exon. The deletion in the third exon indicated that this allele was the *ppo-D1d* allele, previously identified in the wheat D genome progenitor, *Aegilops tauschii* Coss. Additionally, the *ppo-D1d* allele in ‘Fukuhonoka-NIL’ had very low expression, suggesting that this allele is non-functional. We developed a new co-dominant DNA marker for distinguishing the *Ppo-D1a*, *Ppo-D1b* and *ppo-D1d* alleles and demonstrated that F_2_ plants homozygous for the *ppo-D1d* allele exhibited significantly lower grain PPO activity. Additionally, we determined that the *ppo-D1d* allele likely originated from *Ae. tauschii* ssp. *tauschii* (lineage 1) accessions. The *ppo-D1d* allele has not previously been found in common wheat (*Triticum aestivum* L., AABBDD genome), and thus the DNA marker developed in this study will be helpful for introducing this allele in common wheat breeding programs.

## Introduction

The color of wheat flour-based products is an important factor influencing consumer preference. In general, bright and less-dull colors are preferred, and therefore time-dependent darkening and discoloration are undesirable (Morris *et al.* 2000, Siah and Quail 2018). Especially for noodle products, discoloration is a serious issue because these products are preserved in the uncooked state for several days before consumption. There is thus a stronger requirement for minimization of browning than in other products (Baik *et al.* 1995, Morris 2018). To prevent the problem of discoloration, food industries have taken measures including heating, dehydration and inclusion of additives (Ma *et al.* 2023, Zhao *et al.* 2024). However, these methods are disadvantageous with respect to cost and flavor (Fuerst *et al.* 2006, Siah and Quail 2018). Additionally, because the demands for natural products without food additives have increased in recent years, food additives need to be reduced as much as possible. There are two types of discoloration, i.e., enzymatic and non-enzymatic discoloration, enzymatically browning is clearly due to genetic factor mainly determined by polyphenol oxidase (PPO). Therefore, development of wheat cultivars with less grain PPO activity is an important goal in wheat breeding programs.

PPOs are a type of oxidoreductase containing copper, and are classified into tyrosinases (EC 1.14.18.1), catechol oxidases (EC 1.10.3.1), and laccases (EC 1.10.3.2) (Mayer 2006, Zhang 2023). PPOs oxidize mono- or diphenolic compounds to produce quinones, which in turn polymerize with other polyphenols and other compounds, resulting in browning (Anderson and Morris 2003, Baik *et al.* 1994). CuA and CuB, two copper-binding domains characteristic of PPOs, play an important role in their enzymatic function, and the sequence of these domains is highly conserved among species (Van Gelder *et al.* 1997). Additionally, PPOs form multigene families, each of which is functionally differentiated by tissue-specific expression (Demeke and Morris 2002, Jukanti *et al.* 2004). In common wheat (*Triticum aestivum* L., AABBDD genome), *PPOs* form a multigene family consisting of at least 20 genes. Among them, a total of 6–7 genes are specifically expressed in grain and contribute to its discoloration (Jukanti *et al.* 2004, Wold-McGimsey *et al.* 2023); these are classified into two paralogous gene families, *Ppo-1* and *Ppo-2*.

*Ppo-A1* and *Ppo-D1*, which are grouped into the *Ppo-1* paralogues genes, are considered important in wheat breeding programs (Beecher and Skinner 2011, Beecher *et al.* 2012, Zhai *et al.* 2023). These two genes contribute approximately 90% of grain PPO activity (Fuerst *et al.* 2008, Martin *et al.* 2011, Raman *et al.* 2005, Zhang *et al.* 2005), with the *Ppo-A1* gene having the greatest effect on PPO activity, followed by the *Ppo-D1* gene (Nilthong *et al.* 2012, Wang *et al.* 2009). The *Ppo-D1* gene has three alleles, the *Ppo-D1a*, *Ppo-D1b* and *ppo-D1f* alleles, identified in common wheat (He *et al.* 2007, 2009, Hystad *et al.* 2015). The first two of these alleles, *Ppo-D1a* and *Ppo-D1b*, are more frequently found in common wheat (Liu *et al.* 2023, Martin *et al.* 2011, Salaria *et al.* 2018). The *Ppo-D1b* allele is more highly expressed and thus has a greater effect on the grain PPO activity than the *Ppo-D1a* allele (Beecher and Skinner 2011, He *et al.* 2007). The third allele, *ppo-D1f*, carries a nonsense mutation at the first exon and is a non-functional allele (Hystad *et al.* 2015); it was identified in the common wheat line ‘07OR1074’ developed in the U.S. (Hystad *et al.* 2015, Onto 2011). However, no line with this allele has been reported so far. Thus, the null allele of the *Ppo-D1* gene currently has limited utilization in common wheat breeding programs.

‘Fukuhonoka-NIL’ is a very low PPO activity line bred in Japan by backcrossing the Japanese wheat line ‘Fukuhonoka’ with a synthetic hexaploid wheat line having very low grain PPO activity. Recently, we found that the *ppo-A1i* gene in ‘Fukuhonoka-NIL’ had a 3 kb insertion in the second intron and this null allele was originated from tetraploid wheat (Nakamaru *et al.* 2023). However, the *Ppo-D1* of ‘Fukuhonoka-NIL’ has not been characterized. In this study, we attempted to characterize the *Ppo-D1* of ‘Fukuhonoka-NIL’ and develop a new co-dominant DNA marker for high-throughput breeding. We also validated the usefulness of the DNA marker and the effect of the null allele of the *Ppo-D1* on the grain PPO activity on the two F_2_ hybrid populations. Finally, the origin of the null allele of the *Ppo-D1* gene was discussed using phylogenetic analysis.

## Materials and Methods

### Plant materials

All materials used in this study are listed in Supplemental Table 1. The common wheat line ‘Fukuhonoka-NIL’ is a near-isogenic line produced by backcrossing the Japanese wheat line ‘Fukuhonoka’ with a synthetic hexaploid wheat line showing very low grain PPO activity (Chang *et al.* 1997) (Supplemental Fig. 1). The wheat lines ‘Chinese Spring’ and ‘Yumechikara’, which have the *Ppo-D1a* (He *et al.* 2007) and *Ppo-D1b* (Kobayashi *et al.* 2020) alleles, respectively, were used as positive controls. In addition, *Ae. tauschii* var. typica, which was likely the D-genome donor of ‘Fukuhonoka-NIL’ (Supplemental Fig. 1), two lines (accession no. KT120-012 and KT120-013) were obtained from the National BioResource Project (NBRP)-Wheat.

**Fig. 1.**
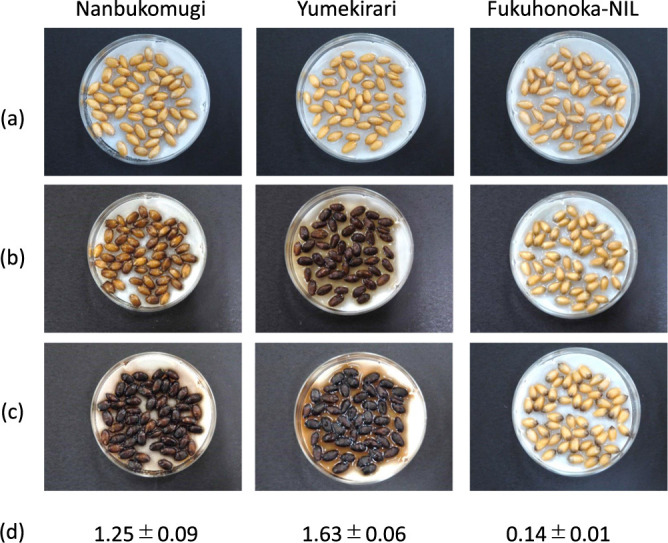
Comparison of the grain PPO activity of parental lines ‘Nanbukomugi’ (*ppo-A1i*, *Ppo-D1a*), ‘Yumekirari’ (*ppo-A1i*, *Ppo-D1b*) and ‘Fukuhonoka-NIL’ (*ppo-A1i*, *ppo-D1d*) of the F_2_ populations. Grain PPO activity harvested under field conditions was evaluated using the phenol test (a, b, c) or the L-DOPA method (d). The incubation times were (a) 0 h, (b) 4 h, (c) 24 h and (d) 1 h. The values determined by the L-DOPA method are shown as the mean ± standard error.

F_2_ segregating populations were derived from a cross between the very-low PPO activity line ‘Fukuhonoka-NIL’ carrying the *ppo-A1i* allele and the medium PPO activity line ‘Nanbukomugi’ carrying the *ppo-A1i* and *Ppo-D1a* alleles, and a cross between ‘Fukuhonoka-NIL’ and the high PPO activity line ‘Yumekirari’ carrying the *ppo-A1i* and *Ppo-D1b* alleles (Fig. 1). Initial crosses were performed in summer 2021 in a greenhouse at the Tohoku National Agricultural Research Center (TARC/NARO). All F_1_ seeds of each cross were planted in the greenhouse in autumn 2022. The heads of each F_1_ plant were harvested and threshed as F_2_ seeds. F_2_ seeds were planted in the greenhouse in autumn 2023 and used to screen individuals carrying homozygous *Ppo-D1a*, *Ppo-D1b* or *ppo-D1d* alleles by a co-dominant DNA marker developed in this study. These lines carrying the *Ppo-A1i* allele were also checked by PPO18Plus (Nakamaru *et al.* 2023). Approximately 30 individuals were analyzed per F_2_ combination, and 4 or 5 individuals per each allele were selected for PPO activity analysis using F_2:3_ seeds.

### Primer design and PCR conditions

The primers used in this study are listed in Supplemental Table 2. The dominant markers for the *Ppo-D1a* allele, PPO16 (He *et al.* 2007) and STS01 (Wang *et al.* 2009), and the dominant marker for the *Ppo-D1b* allele, PPO29 (He *et al.* 2007), were used to test for the presence of these alleles in ‘Fukuhonoka-NIL’. Primers for genotyping, sequencing or RT-PCR of the *Ppo-D1* gene were designed based on the Wheat Chinese Spring IWGSC RefSeq v2.1 genome assembly (https://wheat-urgi.versailles.inra.fr/Seq-Repository/Assemblies), the *Aegilops tauschii* accession AL8/78 genome assembly v5.0 (GenBank BioProject PRJNA341983), or the sequences obtained by direct sequencing of PCR products. Depending on each expected product size, TaKaRa Ex Taq Hot Start Version or TaKaRa LA Taq (TaKaRa Bio, Shiga, Japan) was used for PCR amplifications. PCR was conducted as described by Nakamaru *et al.* (2023).

### Sequencing analysis and phylogenetic analysis

Illumina short reads of ‘Fukuhonoka-NIL’ (Nakamaru *et al.* 2023) were used to detect polymorphisms of the *Ppo-D1* gene in ‘Fukuhonoka-NIL’. The *Ppo-D1* sequence (TraesCS2D468200) from ‘Chinese Spring’ was used as the reference sequence to conduct mapping analysis using the CLC Genomics Workbench 20.0 (Qiagen, Hilden, Germany). The parameters were described in Nakamaru *et al.* (2023). Based on the results, primers were designed to amplify the entire region of the *Ppo-D1* gene. Sequences of PCR products were determined using a PRISM 3130 genetic analyzer (Applied Biosystems, Foster City, CA, USA). The phylogenetic tree was constructed using the CLC Genomics Workbench 20.0 with the UPGMA method. The genetic distances were calculated using the Jukes-Cantor model. Bootstrapping was performed using 1000 replicates.

### Expression analysis

For total RNA extraction, the grains of ‘Fukuhonoka’ and ‘Fukuhonoka-NIL’ were harvested on days after pollination (DAP) 7, 14, 21, and 28. We could not extract RNA from the grains harvested at DAP 35 because the grain samples were fully ripe. Total RNA was extracted using ISOSPIN with DNase Ⅰ (both from Nippon Gene, Toyama, Japan). First-strand cDNA was synthesized from total RNA by reverse-transcription using SuperScriptⅢ (Thermo Fisher Scientific, Waltham, MA) with Oligo(dT)12-18 (Invitrogen, Carlsbad, CA, USA) according to the manufacturer’s instruction. RT-PCR was conducted using two primer sets for RT-PCR (Supplemental Table 2), and the *ACTIN* gene was also amplified as a control. Expression of the *Ppo-D1* gene was normalized to *ACTIN* expression. PCR reactions included 2×PCR buffer for KOD FX (Toyobo, Osaka, Japan), 0.4 μM of dNTPs, 1.0 μM of forward primer, 1.0 μM of reverse primer, 0.5 units of KOD FX polymerase, 1.5 μl of 1st strand cDNA, and dH_2_O to a total volume of 25 μl. An initial denaturation at 94°C for 2 min was followed by 30–35 cycles of PCR (94°C for 10 s, 65°C for 30 s, 68°C for 2 min), and a final extension of 68°C for 7 min. PCR reactions were performed in three replicates for each sample.

### DNA marker analysis

PCR reactions included 2 μl of 5 ×Q solution (Qiagen), 1 μl of CoralLoad PCR Buffer (Qiagen), 0.1 μM of dNTPs, 2.0 μM of forward primer PPO-D1d_F1, 2.0 μM of reverse primer PPO-D1d_R1, 0.05 units of Hotstartaq plus polymerase (Qiagen), 100 ng genomic DNA, and dH_2_O in a total volume of 10 μl. An initial denaturation at 95°C for 5 min was followed by 38 cycles of PCR (94°C for 1 min, 60°C for 1 min, 72°C for 1 min), and a final extension of 72°C for 10 min. PCR was performed using a Veriti 96-well thermal cycler (Thermo Fisher Scientific). PCR products were separated by electrophoresis on 1.8% agarose gels made of agarose for 150–1,500 bp fragment (Nacalai Tesque, Kyoto, Japan) stained with EZ-Vision Three (VWR Life Science, Radnor, PA, USA) and visualized with UV light.

### PPO activity analysis

Grain PPO activity was evaluated for all parental and progeny lines in the F_2_ generations, using the L-DOPA method (AACC International Method 22-85.01) with slight modifications. The L-DOPA solution was made freshly each day: 10 mM L-DOPA (3,4-dihydroxyphenylalanine) was diluted in 50 mM MOPS [3-(N-morpholino) propane sulfonic acid] buffer (Fujifilm, Osaka, Japan) including 0.02% Tween-20 (Sigma-Aldrich, St. Louis, MO, USA). The buffer was adjusted to pH 6.5 using sodium hydroxide. Five grains were put into a 2 mL microcentrifuge tube, and then 1.5 ml of L-DOPA solution was dispensed into the tube. After incubation with shaking for 1 or 2 hours at room temperature, the supernatant absorbance at 475 mm was recorded using a Shimadzu UV-2550 UV spectrophotometer (Shimadzu, Kyoto, Japan). At least two replicates were performed for each line and the mean values were calculated for each allele. For visualizing PPO activity among parental lines which were harvested under field conditions, a phenol test (Wrigley 1976) was performed.

### Statistical analysis

For phenotypic evaluation, the differences among *Ppo-D1* alleles in each F_2_ population were examined by Student’s *t-*test using Microsoft Excel^®^ for Microsoft 365 (2402 version). P values <0.05 were deemed to be statistically significant.

## Results

### Characterization of the *Ppo-D1* allele in Fukuhonoka-NIL

**Fig. 2.**
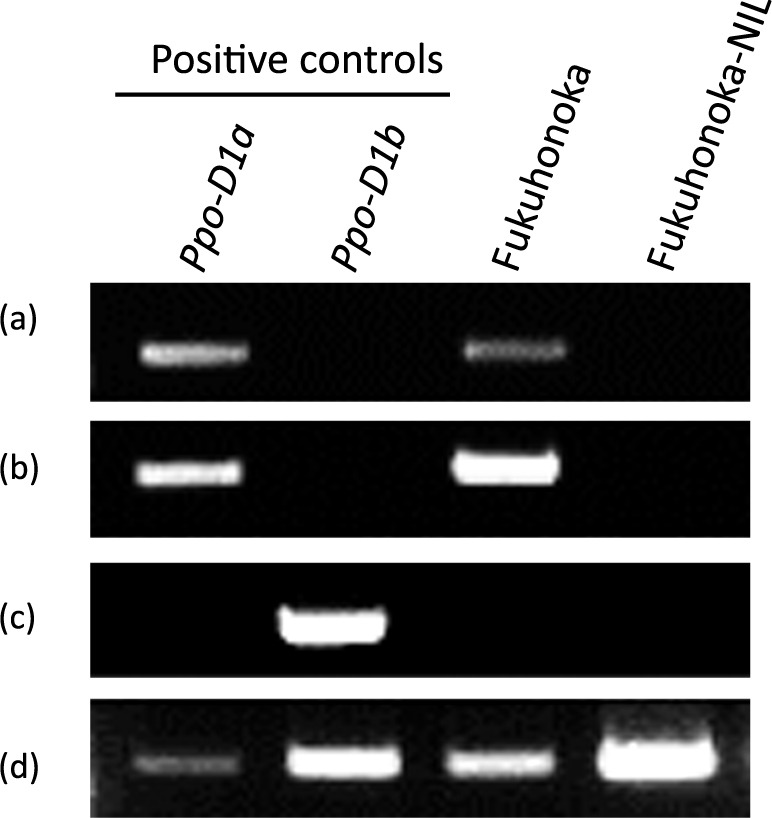
Genotyping of ‘Fukuhonoka-NIL’ using the dominant markers for the *Ppo-D1* allele. ‘Chinese Spring’ and ‘Yumechikara’ were used as positive controls, *Ppo-D1a* and *Ppo-D1b*, respectively. Gel images of (a) STS01 (Wang *et al.* 2009), (b) PPO16 (He *et al.* 2009), (c) PPO29 (He *et al.* 2009) and (d) *ACTIN* (Sun *et al.* 2011). 1.5% agarose gels were used.

Initially, previously reported DNA markers were used to test for the presence of the *Ppo-D1a* and *Ppo-D1b* alleles in ‘Fukuhonoka-NIL’. STS01 (Wang *et al.* 2009) and PPO16 (He *et al.* 2009) are dominant markers for the *Ppo-D1a* allele, and PPO29 (He *et al.* 2009) is a dominant marker for the *Ppo-D1b* allele. *ACTIN* (Sun *et al.* 2011) was used as a positive control. The recurrent parent ‘Fukuhonoka’ was not amplified by PPO29, but PPO16 and STS01 amplified 713 bp and 540 bp products, respectively, indicating that ‘Fukuhonoka’ carries the *Ppo-D1a* allele (Fig. 2a–2c). However, none of the DNA markers could amplify products from ‘Fukuhonoka-NIL’ (Fig. 2a–2c). PPO 29 amplified a 490 bp product from the second exon to the third exon of the *Ppo-D1b* allele (Supplemental Fig. 2). PPO16 amplified a 713 bp product from the second exon to the third exon in lines carrying the *Ppo-D1a* allele (Supplemental Figs. 2, 3). In addition, STS01 was designed from the third exon to the 3′UTR of the *Ppo-D1a* allele to amplify a 510 bp product (Supplemental Figs. 2, 3). The lack of products in ‘Fukuhonoka-NIL’ implies that the *Ppo-D1* gene of ‘Fukuhonoka-NIL’ has some structural mutations or sequence substitutions. We then attempted to amplify the *Ppo-D1* gene from ‘Fukuhonoka-NIL’, but it was difficult to amplify the *Ppo-D1* locus specifically because there is high homology between the *Ppo-D1* and *Ppo-A1* gene in common wheat.

Therefore, we utilized next-generation sequencing (NGS) data obtained from ‘Fukuhonoka-NIL’ (Nakamaru *et al.* 2023), which were mapped to the *Ppo-D1* gene TraesCS2D02G468200. The mapping results suggested that the *Ppo-D1* gene of ‘Fukuhonoka-NIL’ has two structural mutations: a deletion in the third exon and a deletion in the 3′UTR of the *Ppo-D1* gene (Supplemental Fig. 3b). Direct sequencing of PCR products, which were amplified from ‘Fukuhonoka-NIL’ using the primers PPOD1seqF1 and PPOD1seqR1, identified a 73-bp deletion at the third exon and three single nucleotide polymorphisms (SNPs) compared to Chinese Spring (Supplemental Fig. 2). BLAST analysis suggested that the *Ppo-D1* sequence of Fukuhonoka-NIL was almost identical to the *ppo-D1d* allele identified from *Ae. tauchii* accession Y59 (He *et al.* 2009), and the ORF sequence of the *Ppo-D1* of ‘Fukuhonoka-NIL’ has two SNPs: a G>C SNP (+11 bp positions from the ATG site) and a C>G SNP (+328 bp positions from the ATG site) (Supplemental Fig. 2). These SNPs did not result in nonsense mutations and the frameshift mutation occurred at the same location in the sequence as in the accession Y59 (Supplemental Fig. 4); thus, the *Ppo-D1* allele in Fukuhonoka-NIL was considered to be the *ppo-D1d* allele.

### Expression analysis

**Fig. 3.**
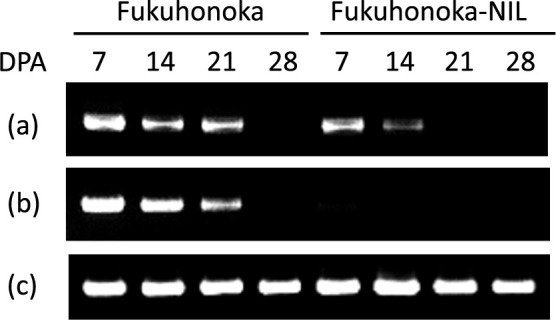
Expression analysis of the *Ppo-D1* gene in ‘Fukuhonoka’ and ‘Fukuhonoka-NIL’ using seeds harvested at DPA 7, 14, 21 and 28. PCR products for (a) the junction site between the first and second exon to the third exon before the 73 bp deletion, (b) the first exon to the 3′UTR, and (c) the internal control gene *ACTIN*. Three replicates were conducted.

To confirm the expression of the *ppo-D1d* allele, semiquantitative RT-PCR analysis in immature seeds of Fukuhonoka (*Ppo-D1a*) and Fukuhonoka-NIL (*ppo-D1d*) were carried out using the *ACTIN* gene as an internal control. Two primer sets for RT-PCR were designed: one was in an exon-exon junction (Fig. 3a) located in the second half of the *Ppo-D1* gene, and the other was between the first exon and the 3′UTR (Fig. 3b) of the *Ppo-D1* gene. The control gene *ACTIN* was expressed in both lines at every stage (Fig. 3c). For ‘Fukuhonoka’ carrying the wild-type allele *Ppo-D1a*, transcripts were detected from DPA 7 to 21 with each primer set of the *Ppo-D1* gene, and no transcript was detected at DPA 28 (Fig. 3a, 3b). For ‘Fukuhonoka-NIL’ carrying the *ppo-D1d* allele, the amount of transcripts when using the primer set prior to the deletion transcripts at DAP 7 was almost equal to that of ‘Fukuhonoka’, but the amount of transcripts of DAP 14 was lower than that of ‘Fukuhonoka’, and there were no transcripts at DAP 21 and 28 (Fig. 3a). Furthermore, using the longer primer set, no transcripts was detected from ‘Fukuhonoka-NIL’ at any stage (Fig. 3b). These results implied that the transcripts level of *ppo-D1d* was lower and full-length transcripts were not produced as they were for the *Ppo-D1a* allele.

### Co-dominant DNA marker development

**Fig. 4.**
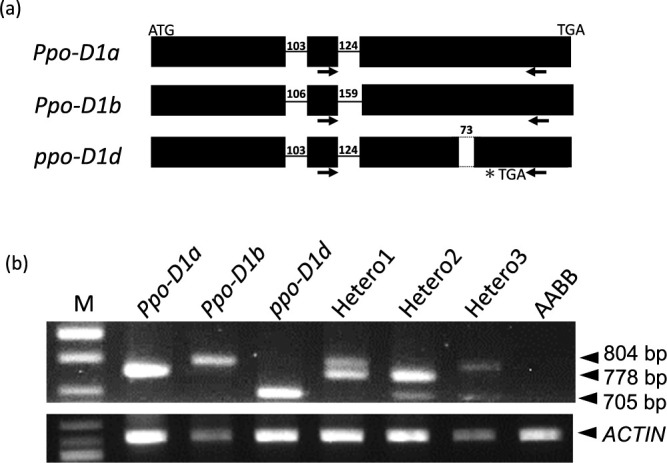
A new co-dominant DNA marker for distinguishing the *ppo-D1d* allele from the *Ppo-D1a* and *Ppo-D1b* alleles. (a) Gene structures of the *Ppo-D1a*, *Ppo-D1b* and *ppo-D1d* alleles. The black boxes are exons and black lines are introns. The numbers above the black lines or boxes indicate the intron or deletion length. The dotted lines represent deletions. Black arrows are the primer position of the co-dominant DNA marker. (b) Gel images of the co-dominant DNA marker and positive control *ACTIN* gene. M: size marker: Quick-Load^®^ 2-Log DNA Ladder (0.1–10 kb); *Ppo-D1a*: ‘Chinese Spring’; *Ppo-D1b*: ‘Yumechikara’; *Ppo-D1d*: ‘Fukuhonoka-NIL’, Hetero1, Hetero2 and Hetero3: artificial heterozygotes composed of the allele pairs *Ppo-D1a/b*, *Ppo-D1a/d* and *Ppo-D1b/d*, respectively; AABB: durum wheat line Mexicali 75. The *Ppo-D1a*, *Ppo-D1b* and *ppo-D1d* alleles produced 778, 804 and 705 bp fragments, respectively. The PCR products were separated on 1.8% gels.

To select the *ppo-D1d* allele, we attempted to develop a new co-dominant DNA marker capable of distinguishing the *Ppo-D1a*, *Ppo-D1b* and *ppo-D1d* alleles. The *Ppo-D1b* allele has a size difference in the second intron compared to the *Ppo-D1a* and the *ppo-D1d* alleles, while the *ppo-D1d* allele has a 73 bp deletion compared to the *Ppo-D1a* and the *Ppo-D1b* alleles (Supplemental Fig. 2). Based on these mutations, we designed a forward primer located at the 5ʹ end of the second intron, and a reverse primer located 53 bp downstream of the 73 bp deletion (Fig. 4a). The new co-dominant marker amplified 778 bp, 804 bp and 705 bp fragments from the *Ppo-D1a*, *Ppo-D1b*, and *ppo-D1d* alleles, and each heterozygous line could be distinguished by agarose-gel based PCR (Fig. 4b). Additionally, this DNA marker did not amplify PCR products from the durum wheat line ‘Mexicali 75’ (AABB), indicating it was a D-genome specific marker.

### Verification of the effect of the *ppo-D1d* allele

In the phenol test, the parental lines of the F_2_ population harvested under field conditions showed that grains of ‘Fukuhonoka-NIL’ did not stain after 4 hours, while those of ‘Nanbukomugi’ stained slightly and those of ‘Yumekirari’ stained darkly (Fig. 1b). Additionally, the L-DOPA test showed that the PPO activity values were higher in the order ‘Yumekirari’, ‘Nanbukomugi’ and ‘Fukuhonoka-NIL’ (Fig. 1d). These parental lines have the *ppo-A1i* allele in common, suggesting that differences in the *Ppo-D1a*, *Ppo-D1b* and the *ppo-D1d* alleles affect the grain PPO activity.

**Fig. 5.**
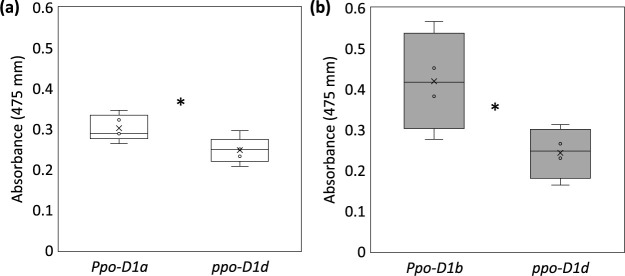
Analysis of the grain PPO activity and the *Ppo-D1* alleles in two F_2_ populations. (a) Nanbukomugi/Fukuhonoka-NIL. (b) Nanbukirari/Fukuhonoka-NIL. Each box shows data of four or five lines carrying each *Ppo-D1* allele. The box represents the 25^th^ and the 75^th^ percentiles and whiskers represent the maximum and minimum value. Statistically significant differences in each F_2_ generation are denoted with an asterisk (*) (Student’s t-test P < 0.05).

To validate the effect of the *ppo-D1d* allele, two F_2_ generations were used to analyze the grain PPO activity. F_2_ generations were produced by crossing Japanese wheat lines ‘Nanbukomugi’ (*ppo-A1i*, *Ppo-D1a*) and ‘Yumekirari’ (*ppo-A1i*, *Ppo-D1b*) with ‘Fukuhonoka-NIL’ (*ppo-A1i*, *ppo-D1d*). Each F_2_ generation segregated the *Ppo-D1* allele on the *ppo-A1i* background, and the homozygous individuals were selected using a newly developed DNA marker for the *ppo-D1d* allele. Although L-DOPA analysis showed a significant difference in the PPO activity values between ‘Yumekirari’ (0.40 ± 0.06) and ‘Fukuhonoka-NIL’ (0.20 ± 0.06), the difference in PPO activity between ‘Nanbukomugi’ (0.24 ± 0.03) and ‘Fukuhonoka-NIL’ (0.20 ± 0.06) was not significant. In the combination of Nanbukomugi/Fukuhonoka-NIL, the mean PPO activity of lines with the *Ppo-D1a* allele was 0.30 ± 0.03 (standard deviation), while that of lines with the *ppo-D1d* allele was 0.25 ± 0.03 (Fig. 5a). In the combination Yumekirari/Fukuhonoka-NIL, the mean PPO activity of lines carrying the *Ppo-D1b* allele was 0.42 ± 0.12, while that of lines carrying the *ppo-D1d* allele was 0.24 ± 0.06 (Fig. 5b). The F_2:3_ lines carrying the *ppo-D1d* allele showed significantly lower PPO activity than the lines carrying the *Ppo-D1a* or *Ppo-D1b* allele (P < 0.05).

### The origin of the *ppo-D1d* allele

Based on the pedigree record of ‘Fukuhonoka-NIL’ (Supplemental Fig. 1), the *Ppo-D1* sequences of two *Ae. tauschii* var. typica accessions, KT120-012 and KT120-013, which might be used to produce a very low PPO activity line, were examined to find the origin of the *ppo-D1d* allele. The sequences of the *Ppo-D1* gene obtained from both accessions were identical and included the 73 bp deletion of the third exon, indicating that they were carrying the *ppo-D1d-like* alleles (Supplemental Fig. 5). Both sequences had one missense SNP on the open reading frame against the sequence in the accession of Y59 (*ppo-D1d*), and this mutation did not result in nonsense mutations. Therefore, the allele of the *Ppo-D1* gene of *Ae. tauschii* accessions KT120-012 and KT120-013 appeared to be the *ppo-D1d* allele. However, the sequence was not perfectly matched to the *ppo-D1d* sequence in ‘Fukuhonoka-NIL’ (Supplemental Fig. 5), suggesting that an *Ae. tauschii* accession closely related to KT120-012 or KT120-013 was likely used as the D-genome donor of ‘Fukuhonoka-NIL’ that was crossed with ‘Mexicali 75’.

**Fig. 6.**
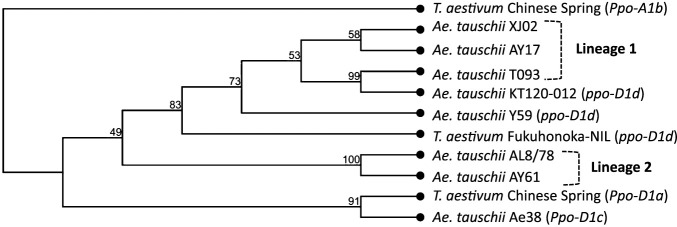
Phylogenetic tree of the ORF sequences of *Ppo-D1* genes in *Ae. tauschii* and *T. aestivum*. The tree was constructed using the software package CLC Genomics Workbench with the UPMEGA method, including the two new alleles identified in this study, the *Ppo-D1a* allele in *T. aestivum* (He *et al.* 2007), the *Ppo-D1c* allele in *Ae. tauschii* (He *et al.* 2009), five *Ae. tauschii* reference genomes (Zhou *et al.* 2021) and the *Ppo-A1b* allele in *T. aestivum* Chinse Spring as the out-group. Bootstraping was performed and the bootstrap values using 1000 replicates are shown.

To obtain further information on the origin of the *ppo-D1d* allele, the *ppo-D1d* sequences in Y59, Fukuhonoka-NIL and KT120-012 obtained in this study were compared with the *Ppo-D1* genes of the available reference genomes of five *Ae. tauschii* accessions (AL8/78, AY17, AY61, T093 and XJ02) (Wang *et al.* 2021, Zhou *et al.* 2021) and the *Ppo-D1c* (GenBank accession: EU371656) identified from *Ae. tauschii* accession Ae38 (He *et al.* 2009). The phylogenetic tree showed that three accessions (AY17, T093, XJ02), representatives of L1, were clustered with Y59, Fukuhonoka-NIL and KT120-012 as the same group, whereas the other accessions (AL8/78, AY61), representatives of lineage 2 (L2), were clustered together as a second group (Fig. 6), suggesting that the *ppo-D1d* allele was present in *Ae. tauschii* L1 accessions.

## Discussion

Introducing null alleles of the *Ppo* genes is critical to develop wheat cultivars with less time-dependent discoloration. DNA marker analysis has played an important role in efficiently selecting favorable alleles in such wheat breeding programs. We focused on the common wheat line ‘Fukuhonoka-NIL’, which exhibits low grain PPO activity and had a null allele of the *Ppo-A1* gene (Nakamaru *et al.* 2023). However, the *Ppo-D1* gene of this line had not been investigated. Although the dominant markers STS01, PPO16 and PPO29 have been used to identify the *Ppo-D1a* allele, which is associated with low PPO activity, no PCR product was amplified using these DNA markers in ‘Fukuhonoka-NIL’ (Fig. 2). In this study, we demonstrated that the *Ppo-D1* sequence of ‘Fukuhonoka-NIL’ holds two structural mutations (Supplemental Fig. 3). Compared to the reference sequences of common wheat and the D-genome donor, the *Ppo-D1* gene (*ppo-D1d*) of ‘Fukuhonoka-NIL’ has an approximately 3 kb deletion in the 3′UTR and a 73 bp deletion in the third exon (Supplemental Figs. 3, 6). These results could explain the reason why the previous DNA markers did not produce any products from the ‘Fukuhonoka-NIL’; both of the reverse primer sites of the STS01 or PPO16 (He *et al.* 2009, Wang *et al.* 2009) were located in a deleted region of the *ppo-D1d* gene of ‘Fukuhonoka-NIL’ (Supplemental Figs. 2, 6).

The *ppo-D1d* allele has been found only in *Ae. tauschii* accessions and was suggested to be a loss-of-function allele due to a frameshift mutation in the third exon (He *et al.* 2009). There are several cases of loss of the gene function caused by frameshift mutations in plants (Emrani *et al.* 2015, Gadjieva *et al.* 2004, Saito and Nakamura 2005, Wang *et al.* 2018). These cases were suggested to be due to nonsense-mediated mRNA decay (NMD) since transcript level were decreased. Similarly, our semiquantitative RT-PCR analysis of grains at maturity showed that no or lower transcripts were detected through seed development compared to the wild-type *Ppo-D1a* allele (Fig. 3a, 3b), which could be caused by NMD. PPO activity analysis in the F_2_ generation also suggested that the *ppo-D1d* alleles resulted in a reduction of grain PPO activity (Fig. 5). Although the effect of the *ppo-D1d* alleles in *Ae. tauschii* was not investigated in this study, they are likely to be null alleles due to the frameshift mutation, as is the case with ‘Fukuhonoka-NIL’. Therefore, these results confirmed that the *ppo-D1d* is a loss-of-function allele.

‘Fukuhonoka-NIL’ is a near-isogenic line developed by utilizing a synthetic wheat showing very low PPO activity. The synthetic wheat was produced by an interspecific cross between the *T. turgidum* subsp. durum ‘Mexicali 75’ and *Ae. tauschii* var. typica (Chang *et al.* 1997) (Supplemental Fig. 1), but this line was not available for this study. Therefore, to clarify the origin of the D-genome donor, *Ae. tauschii* var. typica accessions KT120-012 and KT120-013, which were available among eight lines, were obtained from the NBRP-Wheat. Their origin remains unknown, but they were morphologically classified as *Ae. tauschii* ssp. *tauschii* (lineage 1; L1) (Li *et al.* 2022, Singh *et al.* 2019). We attempted to investigate the origin of the *ppo-D1d* allele by examining two accessions that could be considered D-genome donors of ‘Fukuhonoka-NIL’ and comparison with *Ae. tauschii* reference genomes. Phylogenetic analysis suggested that neither of these accessions obtained from NBRP was the D-genome donor of ‘Fukuhonoka-NIL’, although a closely related accession from ssp. *tauschii* (L1) was possibly used. In addition, this allele was frequently found in L1 accessions rather than L2 (Fig. 6). There are various theories on how common wheat was formed, but it is most likely that tetraploid wheat was hybridized with ssp. *strangulata* (L2) (Li *et al.* 2022, Singh *et al.* 2019, Zhou *et al.* 2021), which is consistent with our results. There were few opportunities to cross with *Ae. tauschii* carrying the *ppo-D1d* allele during wheat establishment, and therefore the *ppo-D1d* allele might not have ever been found in common wheat. Genes derived from the D-genome donors have played an important role in the breeding of common wheat. Examples include the *Glu-D1d* allele for good bread-making properties (Delorean *et al.* 2021) and *Lr21* and *Lr42* for wheat leaf rust resistance (Huang *et al.* 2009, Lin *et al.* 2022). The *ppo-D1d* allele, which is not found in the common wheat germplasm (He *et al.* 2007, Liang *et al.* 2010), can be introduced into breeding programs through synthetic wheat more easily than through direct crosses with *Ae. tauschii* (Kishii 2019).

There are obvious differences in the PPO activity among the parental lines of the F_2_ populations (Fig. 1b, 1d). All these lines have the *ppo-A1i* allele, and thus the PPO activity differences are likely attributable to differences in the *Ppo-D1* alleles. In this study, grains harvested under greenhouse conditions had overall lower PPO activity values than those harvested under field conditions (Figs. 1d, 5). The F_2_ lines carrying the *ppo-D1d* allele, which were harvested under greenhouse conditions, had significantly lower PPO activity than the lines carrying the *Ppo-D1a* or *Ppo-D1b* alleles, with PPO activity declining to the same level as in ‘Fukuhonoka-NIL’, although these PPO activity values tended to be low (Fig. 5). Previous studies have reported that, in addition to the PPO gene, factors such as cis-regulator elements (Liu *et al.* 2020) and environmental conditions (Park *et al.* 2000) also influence PPO activity. Therefore, it is possible that these factors might have influenced PPO activity in the material in this study. However, the new co-dominant DNA marker enabled us to screen lines carrying the *ppo-D1d* allele in segregating populations regardless of environmental conditions. The fact that the lines carrying the null allele had significantly lower grain PPO activity demonstrated the usefulness of this DNA marker.

By utilizing the sequence information of *ppo-D1d*, *Ppo-D1a* and *Ppo-D1b*, we have developed a new co-dominant marker that can distinguish the three alleles by the size differences among amplified products. This marker is specific to the D-genome, which reduces the likelihood of amplifying other homologous genes. Since selection can be performed by agarose gel electrophoresis and does not require construction of a special system such as Kompetitive Allele Specific PCR (KASP), the new marker would be easier to use in breeding programs. Co-dominant DNA markers have the advantage of higher selection efficiency, thereby accelerating the introduction of favorable alleles to commercial varieties by repeated backcrossing. The combination of the newly developed DNA marker and PPO18Plus (Nakamaru *et al.* 2023) will select the null alleles of the *Ppo-A1* and *Ppo-D1* genes simultaneously and contribute to the development of varieties with a low level of grain PPO activity and brighter noodle color.

## Author Contribution Statement

AN and TN designed the study; AN, KK, TN and SI carried out experiments; AN and KH wrote the manuscript; and AN, KH, KK, SI and TN contributed to discussions and critically reviewed the manuscript. All authors read and approved the final manuscript.

## Supplementary Material

Supplemental Figures

Supplemental Tables
