# Supplementary material for: Development and validation of a new co-dominant DNA marker for selecting the null allele of polyphenol oxidase gene Ppo-D1 in common wheat (Triticum aestivum L.)
Source: Breed Sci. 2025 Apr 4;75(2):102–10. doi: 10.1270/jsbbs.24071 (PMC12395200; doi:10.1270/jsbbs.24071)
Supplement: Supplementary file 1 — Supplemental Figures [file 75_102_s1.pdf]

doi: 10.1270/jsbbs.24071

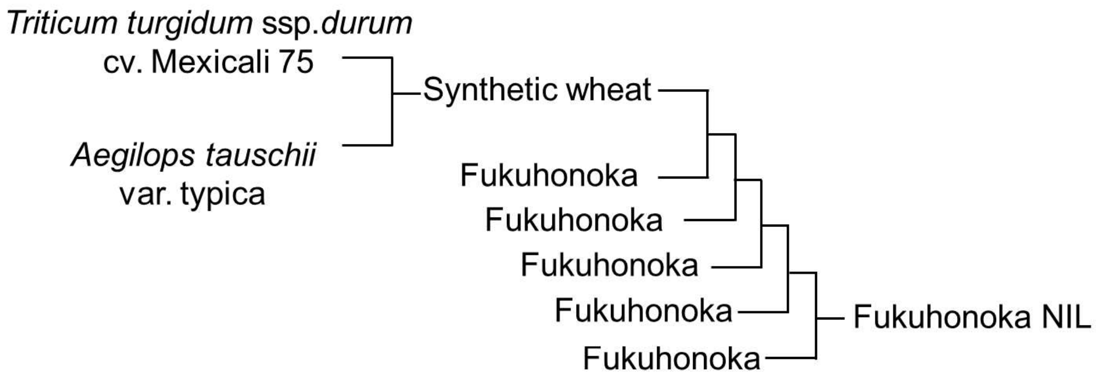

Supplemental Fig. 1. The pedigree record of 'Fukuhonoka-NIL'.

```
                                                                      20                     40                     60      Start codon     80                     100
TraesCS2D02G468200.1;cdna  CACCACAACT AGTGCATTGC ATCCCTAACC CTAAGCGAGC AGCAGCCAGG TAGCAGACAC CGGCAGCCAT GGAGAGCACT CGCGTGCTAC CGAGTGCCAA 100
                 Ppo-D1a  ---------- ---------- ---------- ---------- ---------- ---------- --------AT GGAGAGCAGT CGCGTGCTAC CGAGTGCCAA 32
                 Ppo-D1b  ---------- ---------- ---------- ---------- ---------- ---------- --------AT GGAGAGCAGT CGCATGCCAC TGAGTGCCAC 32
                 Ppo-D1d  ---------- ---------- ---------- ---------- ---------- ---------- --------AT GGAGAGCAGT CGCGTGCTAC CGAGTGCCAA 32
           FukuhonokaNIL  ---------- ---------- ---------- ---------- ---------- ---------- --------AT GGAGAGCACT CGCGTGCTAC CGAGTGCCAA 32

                                     120                    140                    160                    180                    200
TraesCS2D02G468200.1;cdna  CCATCGCATG CCATGCAGCC TCCAAACCTT TCCGCGACGC AACGTTCTCC GTGCCCTTCA CCGCCGCAAG GACGCAAGGC AGCCACGTCG TATCTCAATC 200
                 Ppo-D1a  CCATCGCATG CCATGCAGCC TCCAAACCTT TCCGCGACGC AACGTTCTCC GTGCCCTTCA CCGCCGCAAG GACGCAAGGC AGCCACGTCG TATCTCAATC 132
                 Ppo-D1b  CTCTCGCATG TCATGCAGCC TCCAAACCCT TGCGCGCCGC AACCTTCTCC GTGCCCTTCA CCGCCGGAAG GACGCGAGGC AGCCACGGCG TCTCTCAATC 132
                 Ppo-D1d  CCATCGCATG CCATGCAGCC TCCAAACCTT TCCGCGACGC AACGTTCTCG GTGCCCTTCA CCGCCGCAAG GACGCAAGGC AGCCACGTCG TATCTCAATC 132
           FukuhonokaNIL  CCATCGCATG CCATGCAGCC TCCAAACCTT TCCGCGACGC AACGTTCTCG GTGCCCTTCA CCGCCGCAAG GACGCAAGGC AGCCACGTCG TATCTCAATC 132

                                     220                    240                    260                    280                    300
TraesCS2D02G468200.1;cdna  TCATGCGAGG CGACCGGCGG CGGTCGCGTC GACCGCCGTG AGGTGCTCCT CGGCCTCGGC GGCGCCGCAG CTGCGGGGCT GGCCACGGAC CAAGGTCGAG 300
                 Ppo-D1a  TCATGCGAGG CGACCGGCGG CGGTCGCGTC GACCGCCGTG AGGTGCTCCT CGGCCTCGGC GGCGCCGCAG CTGCGGGGCT GGCCACGGAC CAAGGTCGAG 232
                 Ppo-D1b  TCATGTGAGG CGACCGGCGG CCGCCGCGTC GACCGCCGTG AGGTGCTCCT CGGCCTCGGC GGCGCCGCAG CTGCCGGGCT GGCCACGGAC CAAGGTCGAG 232
                 Ppo-D1d  TCATGCGAGG CGACCGGCGG CGGTCGCGTC GACCGCCGTG AGGTGCTCCT CGGCCTCGGC GGCGCCGCAG CTGCGGGGCT GGCCACGGAC CAAGGTCGAG 232
           FukuhonokaNIL  TCATGCGAGG CGACCGGCGG CGGTCGCGTC GACCGCCGTG AGGTGCTCCT CGGCCTCGGC GGCGCCGCAG CTGCGGGGCT GGCCACGGAC CAAGGTCGAG 232

                                     320                    340                    360                    380                    400
TraesCS2D02G468200.1;cdna  GCGCGATCGC CGCGCCCATC CAGGCCCCGG ACCTCCGCAA CTGCCAAACG CCCGACCTCC CGAACACGCC GCCGGACACC AACTGCTGCC CGACGCCCGG 400
                 Ppo-D1a  GCGCGATCGC CGCGCCCATC CAGGCCCCGG ACCTCCGCAA CTGCCAAACG CCCGACCTCC CGAACACGCC GCCGGACACC AACTGCTGCC CGACGCCCGG 332
                 Ppo-D1b  GCGCGATCGC CGCGCCCATC CAGGCCCCGG ACCTCAGCAA CTGCCAAACG CCCGCCCTCC CGAACACGCC GCCGGATACC AACTGCTGCC CGACGCCCGG 332
                 Ppo-D1d  GCGCGATCGC CGCGCCCATC CAGGCCCCGG ACCTCCGCAA CTGCCAAACG CCCGACCTCC CGAACACGCC GCCGGACACC AACTGCTGCC CGACGCCCGG 332
           FukuhonokaNIL  GCGCGATCGC CGCGCCCATC CAGGCCCCGG ACCTCCGCAA CTGCCAAACG CCCGACCTCC CGAACACGCC GCCGGACACC AACTGCTGCC CGACGGCCGG 332

                                     420                    440                    460                    480                    500
TraesCS2D02G468200.1;cdna  CACCGGCATC ACCGACTTTG AGCTGCCGCC CGCTTCCTCG CCGCTCCGCG TGCGTCCGGC AGCGCACCTG GTGGACGCGG AGTACCTGGC CAAGTACGAG 500
                 Ppo-D1a  CACCGGCATC ACCGACTTTG AGCTGCCGCC CGCTTCCTCG CCGCTCCGCG TGCGTCCGGC AGCGCACCTG GTGGACGCGG AGTACCTGGC CAAGTACGAG 432
                 Ppo-D1b  CACCGGCATC ACCGACTTCG AGCTGCCGCC CGCCTCCTCG CCGCTCCGCG TGCGTCCGGC CGCGCACCTG GTGGACGCGG AGTACCTGGC CAAGTACGAG 432
                 Ppo-D1d  CACCGGCATC ACCGACTTTG AGCTGCCGCC CGCTTCCTCG CCGCTCCGCG TGCGTCCGGC AGCGCACCTG GTGGACGCGG AGTACCTGGC CAAGTACGAG 432
           FukuhonokaNIL  CACCGGCATC ACCGACTTTG AGCTGCCGCC CGCTTCCTCG CCGCTCCGCG TGCGTCCGGC AGCGCACCTG GTGGACGCGG AGTACCTGGC CAAGTACGAG 432

                                     520                    540                    560                    580                    600
TraesCS2D02G468200.1;cdna  AGGGCCGTGG CGCTCATGAA GCAGCTGCCT GCCGATGACC CGCGCAGCTT CGAGCAGCAG TGGCACGTGC ACTGCGCCTA CTGCGACGCC GCCTTCGACC 600
                 Ppo-D1a  AGGGCCGTGG CGCTCATGAA GCAGCTGCCT GCCGATGACC CGCGCAGCTT CGAGCAGCAG TGGCACGTGC ACTGCGCCTA CTGCGACGCC GCCTTCGACC 532
                 Ppo-D1b  AGGGCCGTGG CGCTCATGAA GCAGCTGCCC GCCGATGACC CCCGCAGCTT CGAGCAGCAG TGGCACGTGC ACTGTGCCTA CTGCGACGCC GCCTACGACC 532
                 Ppo-D1d  AGGGCCGTGG CGCTCATGAA GCAGCTGCCC GCCGATGACC CGCGCAGCTT CGAGCAGCAG TGGCACGTGC ACTGCGCCTA CTGCGACGCC GCCTTCGACC 532
           FukuhonokaNIL  AGGGCCGTGG CGCTCATGAA GCAGCTGCCC GCCGATGACC CGCGCAGCTT CGAGCAGCAG TGGCACGTGC ACTGCGCCTA CTGCGACGCC GCCTTCGACC 532

                                     620                    640                    660                    680                    700
TraesCS2D02G468200.1;cdna  AGGTCGGGTT CCCGGACCTT GAGATCCAGG TGCACAACTG CTGGCTCTTC TTCCCATGGC ACAGGTTC-- ---------- ---------- ---------- 668
                 Ppo-D1a  AGGTCGGGTT CCCGGACCTT GAGATCCAGG TGCACAACTG CTGGCTCTTC TTCCCATGGC ACAGGTTCGT AT----GGTC AATGGGTTAA TGGGTGCGAC 628
                 Ppo-D1b  AGGTCGGGTT CCCGGACCTG GAGCTCCAGA TACACAACTG CTGGCTCTTC TTCCCATGGC ACAGGTTCGT ATATATGGTC AATGGGTTA- TGGGTGAGAC 631
                 Ppo-D1d  AGGTCGGGTT CCCGGACCTG GAGATCCAGG TGCACAACTG CTGGCTCTTC TTCCCATGGC ACAGGTTCGT AT----GGTC AATGGGTTA- TGGGTGCGAC 627
           FukuhonokaNIL  AGGTCGGGTT CCCGGACCTG GAGATCCAGG TGCACAACTG CTGGCTCTTC TTCCCATGGC ACAGGTTCGT AT----GGTC AATGGGTTA- TGGGTGCGAC 627

                                     720                    740                    760                    780                    800
TraesCS2D02G468200.1;cdna  ---------- ---------- ---------- ---------- ---------- ---------- ---------- -----TACGT CTACTTCCAC GAGAGGATCC 693
                 Ppo-D1a  GACTTGCACC TTTCTGTGCT GAACCTCAAG GAGCCGTCAC TTGTCCCTGC GTGCGTTTGC TGAACGTGCA GGTTCTACGT CTACTTCCAC GAGAGGATCC 728
                 Ppo-D1b  GACCTGCACC TTTCTGTGCT GAACCTCAAG GAGCCGTCAC TTGTCCCTGC GTGCGTTTGC TGAACGTGCA GGTTCTACCT CTACTTCCAC GAGAGGATCC 731
                 Ppo-D1d  GACTTGCACC TTTCTGTGCT GAACCTCAAG GAGCCGTCAC TTGTCCCTGC GTGCGTTTGC TGAACGTGCA GGTTCTACGT CTACTTCCAC GAGAGGATCC 727
           FukuhonokaNIL  GACTTGCACC TTTCTGTGCT GAACCTCAAG GAGCCGTCAC TTGTCCCTGC GTGCGTTTGC TGAACGTGCA GGTTCTACGT CTACTTCCAC GAGAGGATCC 727

                                     820                    840                    860   →Forward primer PPO29F 880              900
TraesCS2D02G468200.1;cdna  TCGGCAAGCT CATCGGCGAC GACACCTTCG CGCTGCCCTT CTGGAACTGG GACGCGCCGG CCGGCATGAC GCTGCCGGCG ATCTACGCCA ACAGGTCGTC 793
                 Ppo-D1a  TCGGCAAGCT CATCGGCGAC GACACCTTCG CGCTGCCCTT CTGGAACTGG GACGCGCCGG CCGGCATGAC GCTGCCGGCG ATCTACGCCA ACAGGTCGTC 828
                 Ppo-D1b  TCGGCAAGCT CATCGGCGAC GACACCTTCG CGCTGCCGTT CTGGAACTGG GACGCGCCGG CCGGCATGAA GCTGCCGGTC ATCTACGCCA ACAGATCGTC 831
                 Ppo-D1d  TCGGCAAGCT CATCGGCGAC GACACCTTCG CGCTGCCCTT CTGGAACTGG GACGCGCCGG CCGGCATGAC GCTGCCGGCG ATCTACGCCA ACAGGTCGTC 827
           FukuhonokaNIL  TCGGCAAGCT CATCGGCGAC GACACCTTCG CGCTGCCCTT CTGGAACTGG GACGCGCCGG CCGGCATGAC GCTGCCGGCG ATCTACGCCA ACAGGTCGTC 827

                                     920                    940 →Forward primer PPO16F 960                   980                  1,000
TraesCS2D02G468200.1;cdna  GCCGCTCTAC GACGAGAGGC GCGACCCCGC CCACCAGCCG CCGGTGCTGA CCGACCTTGA CTCCAGTGGG ACCGACGCCA ATATCCCAAG AGACCAGCAG 893
                 Ppo-D1a  GCCGCTCTAC GACGAGAGGC GCGACCCCGC CCACCAGCCG CCGGTGCTGA CCGACCTTGA CTCCAGTGGG ACCGACGCCA ATATCCCAAG AGACCAGCAG 928
                 Ppo-D1b  GCCGCTCTAC GACGAGAGGC GCGACCCCGC CCACCAGCCG CCGGTACTGG TCGACCTTGA CTACAGTGGG ACCGACGCCA ACATCCCAAG AGACCAGCAG 931
                 Ppo-D1d  GCCGCTCTAC GACGAGAGGC GCGACCCCGC CCACCAGCCG CCGGTGCTGA TCGACCTTGA CTCCAGTGGG ACCGACGCCA ATATCCCAAG AGACCAGCAG 927
           FukuhonokaNIL  GCCGCTCTAC GACGAGAGGC GCGACCCCGC CCACCAGCCG CCGGTGCTGA TCGACCTTGA CTCCAGTGGG ACCGACGCCA ATATCCCAAG AGACCAGCAG 927

                                   1,020                  1,040                  1,060                  1,080                  1,100
TraesCS2D02G468200.1;cdna  ATCGATCAGA ACCTCAAGAT CATGTACCGC CAG------- ---------- ---------- ---------- ---------- ---------- ---------- 926
                 Ppo-D1a  ATCGATCAGA ACCTCAAGAT CATGTACCGC CAGGCCAGTA GTACTAATTA ACAACCTCAA GAATCCCTAA A--------- ------CAAA A--------- 1004
                 Ppo-D1b  ATCGATGAGA ACCTCAAGAT CATGTACCGC CAGGCCAGTA TTTCTAATTA ACAACCTCAA GAATCCCTAA AAATATTTAG CAACTTCAAA AATATTGGGG 1031
                 Ppo-D1d  ATCGATCAGA ACCTCAAGAT CATGTACCGC CAGGCCAGTA GTACTAATTA ACAACCTCAA GAATCCCTAA A--------- ------CAAA A--------- 1003
           FukuhonokaNIL  ATCGATCAGA ACCTCAAGAT CATGTACCGC CAGGCCAGTA GTACTAATTA ACAACCTCAA GAATCCCTAA A--------- ------CAAA A--------- 1003

                                   1,120                  1,140                  1,160                  1,180                  1,200
TraesCS2D02G468200.1;cdna  ---------- ---------- ---------- ---------- ---------- ---------- ---------- ---------- ---------- ---ATGATTT 933
                 Ppo-D1a  -------TGA GTAGCAACTT CAAAAATATT GTTAAGGTAA CCACAGAACC ACTGGTCATG AAAT-----A ACACAAATGT ACGTACGCAC CAGATGATTT 1092
                 Ppo-D1b  TTTGTTTTGC GAAACAACTT CAAAAACGTT GTTACG-TAA CCACAGAACC ACTGGTCATT AAATTAAATA ACACAAATGT ACATACACAT CAGATGATTT 1130
                 Ppo-D1d  -------TGA GTAGCAACTT CAAAAATATT GTTAAGGTAA CCACAGAACC ACTGGTCATG AAAT-----A ACACAAATGT ACGTACGCAC CAGATGATTT 1091
           FukuhonokaNIL  -------TGA GTAGCAACTT CAAAAATATT GTTAAGGTAA CCACAGAACC ACTGGTCATG AAAT-----A ACACAAATGT ACGTACGCAC CAGATGATTT 1091

                                   1,220                  1,240                  1,260                  1,280                  1,300
TraesCS2D02G468200.1;cdna  CGGACGCGAA GAAGACGCTG CTGTTCCTGG GACAGCCGTA CCGCGCCGGC GACCAGCCGG ACCCGGGCGC GGGCTCCCTG GAGAACGTGC CGCACGGCAC 1033
                 Ppo-D1a  CGGACGCGAA GAAGACGCTG CTGTTCCTGG GACAGCCGTA CCGCGCCGGC GACCAGCCGG ACCCGGGCGC GGGCTCCCTG GAGAACGTGC CGCACGGCAC 1192
                 Ppo-D1b  CAAACGCGAA GAAGACGCTG CTGTTCCTGG GACAGCCGTA CCGCGCCGGC GACCAGCCGG ACCCGGGCGC GGGCTCCGTG GAGAACGTGC CGCACGGCCC 1230
                 Ppo-D1d  CGGACGCGAA GAAGACGCTG CTGTTCCTGG GACAGCCGTA CCGCGCCGGC GACCAGCCGG ACCCGGGCGC GGGCTCCCTG GAGAACGTGC CGCACGGCAC 1191
           FukuhonokaNIL  CGGACGCGAA GAAGACGCTG CTGTTCCTGG GACAGCCGTA CCGCGCCGGC GACCAGCCGG ACCCGGGCGC GGGCTCCCTG GAGAACGTGC CGCACGGCAC 1191

                                   1,320                  1,340 Reverse primer PPO29R← 1,360                1,380                  1,400
TraesCS2D02G468200.1;cdna  GGTCCACGTC TGGACTGGCG ACCCGGCGCA GCCGAACTTG GAGGACATGG GCAACTTCTT CTCGGCGGCG CGCGACCCCA TCTTCTTCGC GCACCACGGC 1133
                 Ppo-D1a  GGTCCACGTC TGGACTGGCG ACCCGGCGCA GCCGAACTTG GAGGACATGG GCAACTTCTT CTCGGCGGCG CGCGACCCCA TCTTCTTCGC GCACCACGGC 1292
                 Ppo-D1b  GGTGCATAAC TGGACAGGCG ACCCAAGGCA GCCGAACGGC GAGGACATGG GCAACTTCTA CTCGGCGGCG CGCGACCCCA TCTTCTTCGC GCACCACGGC 1330
                 Ppo-D1d  GGTCCACGTC TGGACTGGCG ACCCGGCGCA GCCGAACTTG GAGGACATGG GCAACTTCTT CTCGGCGGCG CGCGACCCCA TCTTCTTCGC GCACCACGGC 1291
           FukuhonokaNIL  GGTCCACGTC TGGACTGGCG ACCCGGCGCA GCCGAACTTG GAGGACATGG GCAACTTCTT CTCGGCGGCG CGCGACCCCA TCTTCTTCGC GCACCACGGC 1291

                                   1,420                  1,440                  1,460                  1,480                  1,500
TraesCS2D02G468200.1;cdna  AACATCGACC GCCTGTGGCA CGTCTGGCGC CGCCTCCGCC CGAGCAACAC CGACTTCACC GACCCCGACT GGCTCGACGC CGCCTTCCTC TTCTACGACG 1233
                 Ppo-D1a  AACATCGACC GCCTGTGGCA CGTCTGGCGC CGCCTCCGCC CGAGCAACAC CGACTTCACC GACCCCGACT GGCTCGACGC CGCCTTCCTC TTCTACGACG 1392
                 Ppo-D1b  AACATCGACC GCCTGTGGCA CGTCTGGCGC GGCCTCCGCC CGAGCAACAC CGACTTCACC GACCCCGACT GGCTCGACGC CGGCTTCCTC TTCTACGACG 1430
                 Ppo-D1d  AACATCGACC GCCTGTGGCA CGTCTGGCGC CGCCTCCGCC CGAGCAACAC CGACTTCACC GACCCCGACT GGCTCGACGC CGCCTTCCTC TTCTACGACG 1391
           FukuhonokaNIL  AACATCGACC GCCTGTGGCA CGTCTGGCGC CGCCTCCGCC CGAGCAACAC CGACTTCACC GACCCCGACT GGCTCGACGC CGCCTTCCTC TTCTACGACG 1391

                                   1,520                  1,540                  1,560                  1,580                  1,600
TraesCS2D02G468200.1;cdna  AGGAGGCCCG CCCCGTGCGC GTGCGCGTCC GGGACTGCCT CGACCCGGCC GCGCTGCGGT ACACGTACCA GGACGTCGGC CTGCCGTGGC TCAACGCCAG 1333
                 Ppo-D1a  AGGAGGCCCG CCCCGTGCGC GTGCGCGTCC GGGACTGCCT CGACCCGGCC GCGCTGCGGT ACACGTACCA GGACGTCGGC CTGCCGTGGC TCAACGCCAG 1492
                 Ppo-D1b  AGGAGGCCCG CCCCGTGCGC GTGCGCGTCC GGGACTGCCT CGACCCGGCC GCGCTGCGGT ACACGTACCA GGACGTCGGC CTGCCGTGGC TCAACGCCAG 1530
                 Ppo-D1d  AGGAGGCCCG CCCCGTGCGC GTGCGCGTCC GGGACTGCCT CGACCCGGCC GCGCTGCGGT ACACGTACCA GGACGTCGGC CTGCCGTGGC TCAACGCCAG 1491
           FukuhonokaNIL  AGGAGGCCCG CCCCGTGCGC GTGCGCGTCC GGGACTGCCT CGACCCGGCC GCGCTGCGGT ACACGTACCA GGACGTCGGC CTGCCGTGGC TCAACGCCAG 1491

                                   1,620                  1,640                  1,660            Reverse primer PPO16R←      1,700
TraesCS2D02G468200.1;cdna  GCCGGCCAAG GCGTCCGGCG GGACGCCGGC GCCCGCCACA ACCGGTACCC TCCCTGCCAC CCTGGACAGG ACCATACGGG TGACGGTGAC GAGGCCCAGA 1433
                 Ppo-D1a  GCCGGCCAAG GCGTCCGGCG GGACGCCGGC GCCCGCCACA ACCGGTACCC TCCCTGCCAC CCTGGACAGG ACCATACGGG TGACGGTGAC GAGGCCCAGA 1592
                 Ppo-D1b  GCCAGCCAAG GCGTCCGGCG GGACGCCGGC GCCCGCCACC ACCGGTACGC TCCCTGCCAC CCTGGACAGG ACCATACGGG TGACGGTGAC GAGGCCCAGA 1630
                 Ppo-D1d  GCCGCCCAAG GCGTCCGGCG GGACGCCGGC GCCCGCCACA ACCGG----- ---------- ---------- ---------- ---------- ---------- 1536
           FukuhonokaNIL  GCCGCCCAAG GCGTCCGGCG GGACGCCGGC GCCCGCCACA ACCGG----- ---------- ---------- ---------- ---------- ---------- 1536
                                                                             A 73 bp deletion
```

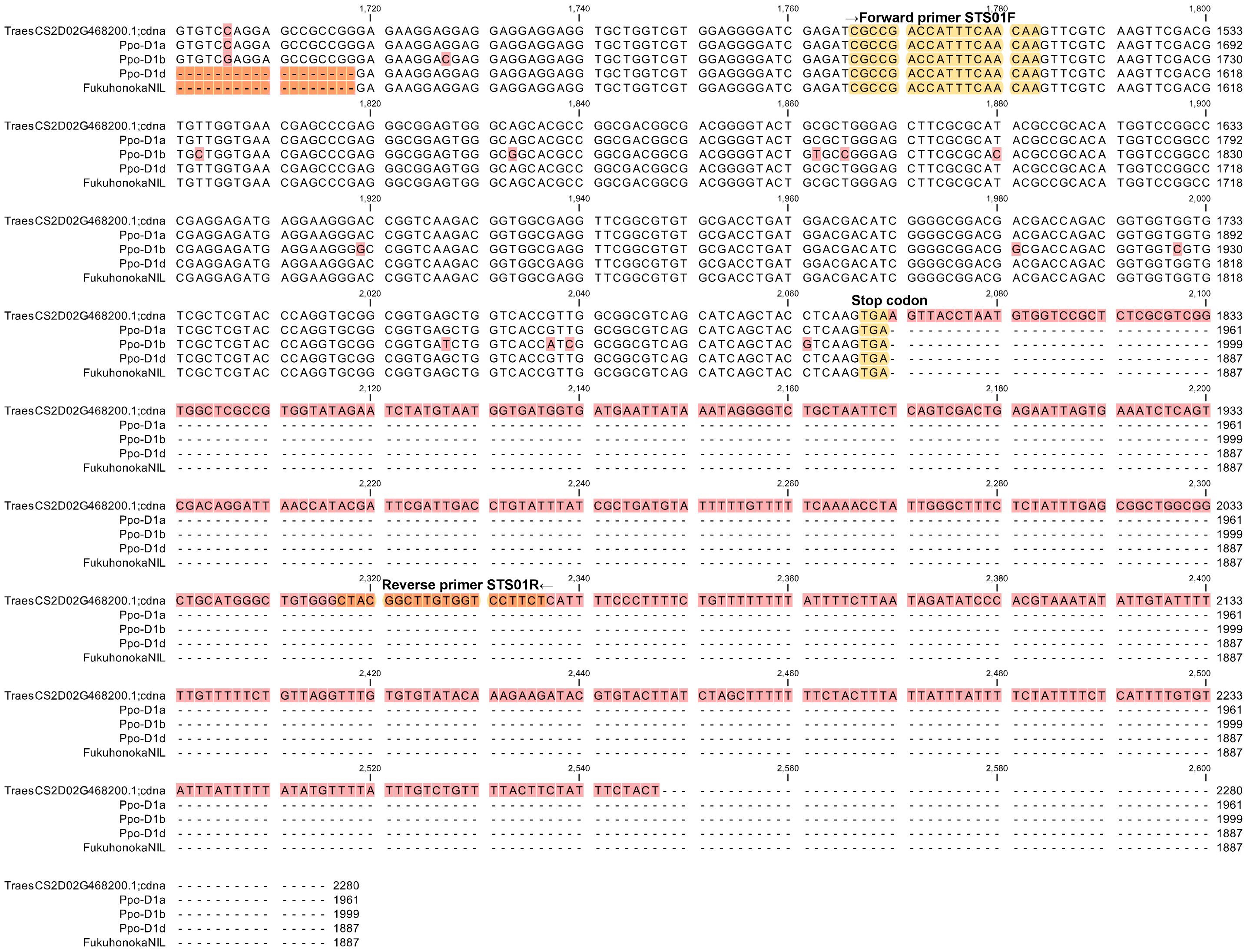

Supplemental Fig. 2. Alignment of the cDNA sequence of 'Chinse Spring' (TraesCS2D468200) and ORF sequences obtained from *Ppo-D1a* (GenBank accession no. EF070149), *Ppo-D1b* (GenBank accession no. EF070150), *ppo-D1d* (GenBank accession no. EU371657) and 'Fukuhonoka-NIL' (GenBank accession no. LC843431). Start and stop codons and primer positions are shown in yellow.

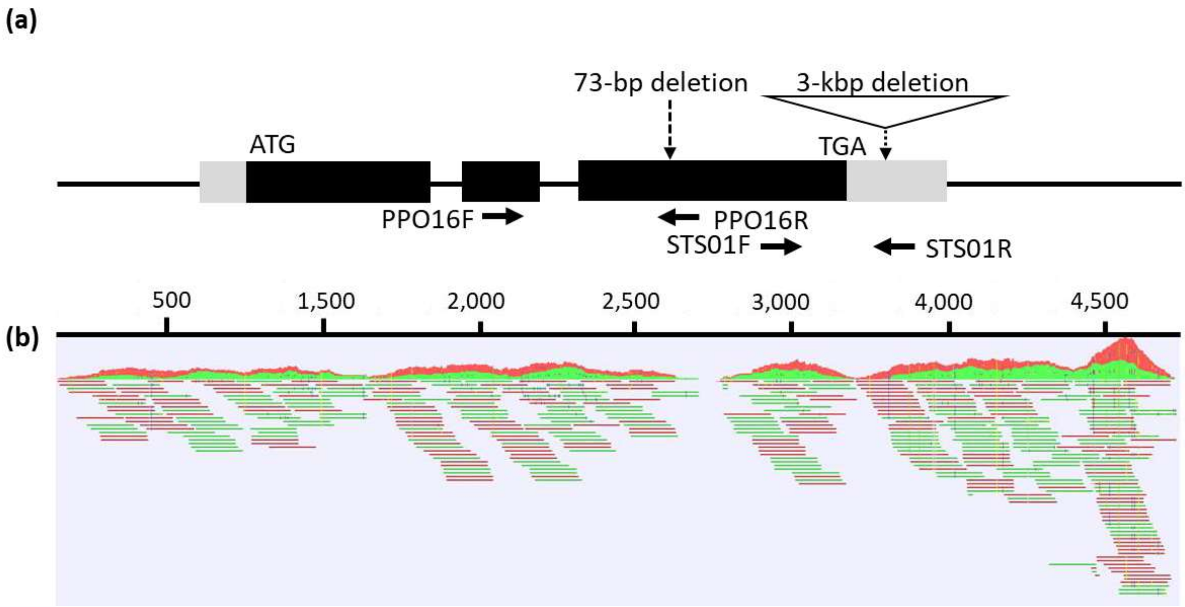

Supplemental Fig. 3. (a) 2D reference sequence around the *Ppo-D1* gene (TraesCS2D02G468200). The black line indicates the 2D reference sequence (IWGSC reference sequence, version 1.0). Black boxes indicate exons. Gray boxes indicate 5'UTR or 3'UTR. Black arrows indicate primers. (b) Mapping result of the 2D reference sequence around TraesCS2D02G468200 using NGS short reads from 'Fukuhonoka-NIL' by CLC Genomics Workbench. Red and green bars indicate reads mapped to sense and anti-sense strands, respectively.

```
                                  20                                      40                                      60                                      80                                     100
                                   |                                       |                                       |                                       |                                       |
Ppo-D1a         MESTRVLPSA NHRMPCSLQT FPRRNVLRAL HRRKDARQPR RISISCEATG GGRVDRREVL LGLGGAAAAG LATDQGRGAI AAPIQAPDLR NCQTPDLPNT 100
ppo-D1d         ...S...... .......... .......G.. .......... .......... .......... .......... .......... .......... .......... 100
Fukuhonoka-NIL  .......... .......... .......G.. .......... .......... .......... .......... .......... .......... .......... 100
KT120-012       .......... .......... .......G.. .......... .......... .......... .......... .......... .......... .......... 100
                                 120                                     140                                     160                                     180                                     200
                                   |                                       |                                       |                                       |                                       |
Ppo-D1a         PPDTNCCPTP GTGITDFELP PASSPLRVRP AAHLVDAEYL AKYERAVALM KQLPADDPRS FEQQWHVHCA YCDAAFDQVG FPDLEIQVHN CWLFFPWHRF 200
ppo-D1d         .......... .......... .......... .......... .......... .......... .......... .......... .......... .......... 200
Fukuhonoka-NIL  .........A .......... .......... .......... .......... .......... .......... .......... .......... .......... 200
KT120-012       .......... .......... .......... .......... .......... .......... .......... .......... .......... .......... 200
                                 220                                     240                                     260                                     280                                     300
                                   |                                       |                                       |                                       |                                       |
Ppo-D1a         YVYFHERILG KLIGDDTFAL PFWNWDAPAG MTLPAIYANR SSPLYDERRD PAHQPPVLTD LDSSGTDANI PRDQQIDQNL KIMYRQMISD AKKTLLFLGQ 300
ppo-D1d         .......... .......... .......... .......... .......... ........I. .......... .......... .......... .......... 300
Fukuhonoka-NIL  .......... .......... .......... .......... .....N.... ........I. .......... .......... .......... .......... 300
KT120-012       .......... .......... .......... .......... .......... ........I. .......... .......... .......... .......... 300
                                 320                                     340                                     360                                     380                                     400
                                   |                                       |                                       |                                       |                                       |
Ppo-D1a         PYRAGDQPDP GAGSLENVPH GTVHVWTGDP AQPNLEDMGN FFSAARDPIF FAHHGNIDRL WHVWRRLRPS NTDFTDPDWL DAAFLFYDEE ARPVRVRVRD 400
ppo-D1d         .......... .......... .......... .......... .......... .......... .......... .......... .......... .......... 400
Fukuhonoka-NIL  .......... .......... .......... .......... .......... .......... .......... .......... .......... .......... 400
KT120-012       .......... .......... .......... .......... .......... .......... .......... .......... .......... .......... 400
                                 420                                     440                                     460                                     480                                     500
                                   |                                       |                                       |                                       |                                       |
Ppo-D1a         CLDPAALRYT YQDVGLPWLN ARPAKASGGT PAPATTGTLP ATLDRTIRVT VTRPRVSRSR REKEEEEEVL VVEGIEIADH FNKFVKFDVL VNEPEGGVGS 500
ppo-D1d         .......... .......... ...P...... .......RRR RRRRCWSWRG SRS.TI.T.S SSSTCW*--- ---------- ---------- ---------- 467
Fukuhonoka-NIL  .......... .......... ...P...... .......RRR RRRRCWSWRG SRS.TI.T.S SSSTCW*--- ---------- ---------- ---------- 467
KT120-012       .......... .......... ...P...... .......RRR RRRRCWSWRG SRS.TI.T.S SSSTCW*--- ---------- ---------- ---------- 467
                                 520                                     540                                     560
                                   |                                       |                                       |
Ppo-D1a         TPATATGYCA GSFAHTPHMV RPEEMRKGPV KTVARFGVCD LMDDIGADDD QTVVVSLVPR CGGELVTVGG VSISYLK* 578
ppo-D1d         ---------- ---------- ---------- ---------- ---------- ---------- ---------- -------- 467
Fukuhonoka-NIL  ---------- ---------- ---------- ---------- ---------- ---------- ---------- -------- 467
KT120-012       ---------- ---------- ---------- ---------- ---------- ---------- ---------- -------- 467
```

Supplemental Fig. 4. Alignment of deduced amino-acid sequences among four *Ppo-D1* alleles.

GenBank accession numbers: *Ppo-D1a* (ABK62803), *ppo-D1d* (ACB12087), Fukuhonoka-NIL (BFT21354), and KT120-012 (BFT21355). Dots indicate identical amino acids.

```
                                      20                    40                    60                    80                   100
                                       |                     |                     |                     |                     |
Ppo-D1d        ATGGAGAGCA GTCGCGTGCT ACCGAGTGCC AACCATCGCA TGCCATGCAG CCTCCAAACC TTTCCGCGAC GCAACGTTCT CGGTGCCCTT CACCGCCGCA 100
FukuhonokaNIL  ATGGAGAGCA CTCGCGTGCT ACCGAGTGCC AACCATCGCA TGCCATGCAG CCTCCAAACC TTTCCGCGAC GCAACGTTCT CGGTGCCCTT CACCGCCGCA 100
KT-012         ATGGAGAGCA CTCGCGTGCT ACCGAGTGCC AACCATCGCA TGCCATGCAG CCTCCAAACC TTTCCGCGAC GCAACGTTCT CGGTGCCCTT CACCGCCGCA 100
KT-013         ATGGAGAGCA CTCGCGTGCT ACCGAGTGCC AACCATCGCA TGCCATGCAG CCTCCAAACC TTTCCGCGAC GCAACGTTCT CGGTGCCCTT CACCGCCGCA 100
                                     120                   140                   160                   180                   200
                                       |                     |                     |                     |                     |
Ppo-D1d        AGGACGCAAG GCAGCCACGT CGTATCTCAA TCTCATGCGA GGCGACCGGC GGCGGTCGCG TCGACCGCCG TGAGGTGCTC CTCGGCCTCG GCGGCGCCGC 200
FukuhonokaNIL  AGGACGCAAG GCAGCCACGT CGTATCTCAA TCTCATGCGA GGCGACCGGC GGCGGTCGCG TCGACCGCCG TGAGGTGCTC CTCGGCCTCG GCGGCGCCGC 200
KT-012         AGGACGCAAG GCAGCCACGT CGTATCTCAA TCTCATGCGA GGCGACCGGC GGCGGTCGCG TCGACCGCCG TGAGGTGCTC CTCGGCCTCG GCGGCGCCGC 200
KT-013         AGGACGCAAG GCAGCCACGT CGTATCTCAA TCTCATGCGA GGCGACCGGC GGCGGTCGCG TCGACCGCCG TGAGGTGCTC CTCGGCCTCG GCGGCGCCGC 200
                                     220                   240                   260                   280                   300
                                       |                     |                     |                     |                     |
Ppo-D1d        AGCTGCGGGG CTGGCCACGG ACCAAGGTCG AGGCGCGATC GCCGCGCCCA TCCAGGCCCC GGACCTCCGC AACTGCCAAA CGCCCGACCT CCCGAACACG 300
FukuhonokaNIL  AGCTGCGGGG CTGGCCACGG ACCAAGGTCG AGGCGCGATC GCCGCGCCCA TCCAGGCCCC GGACCTCCGC AACTGCCAAA CGCCCGACCT CCCGAACACG 300
KT-012         AGCTGCGGGG CTGGCCACGG ACCAAGGTCG AGGCGCGATC GCCGCGCCCA TCCAGGCCCC GGACCTCCGC AACTGCCAAA CGCCCGACCT CCCGAACACG 300
KT-013         AGCTGCGGGG CTGGCCACGG ACCAAGGTCG AGGCGCGATC GCCGCGCCCA TCCAGGCCCC GGACCTCCGC AACTGCCAAA CGCCCGACCT CCCGAACACG 300
                                     320                   340                   360                   380                   400
                                       |                     |                     |                     |                     |
Ppo-D1d        CCGCCGGACA CCAACTGCTG CCCGACGCCC GGCACCGGCA TCACCGACTT TGAGCTGCCG CCCGCTTCCT CGCCGCTCCG CGTGCGTCCG GCAGCGCACC 400
FukuhonokaNIL  CCGCCGGACA CCAACTGCTG CCCGACGGCC GGCACCGGCA TCACCGACTT TGAGCTGCCG CCCGCTTCCT CGCCGCTCCG CGTGCGTCCG GCAGCGCACC 400
KT-012         CCGCCGGACA CCAACTGCTG CCCGACGCCC GGCACCGGCA TCACCGACTT TGAGCTGCCG CCCGCTTCCT CGCCGCTCCG CGTGCGTCCG GCAGCGCACC 400
KT-013         CCGCCGGACA CCAACTGCTG CCCGACGCCC GGCACCGGCA TCACCGACTT TGAGCTGCCG CCCGCTTCCT CGCCGCTCCG CGTGCGTCCG GCAGCGCACC 400
                                     420                   440                   460                   480                   500
                                       |                     |                     |                     |                     |
Ppo-D1d        TGGTGGACGC GGAGTACCTG GCCAAGTACG AGAGGGCCGT GGCGCTCATG AAGCAGCTGC CCGCCGATGA CCCGCGCAGC TTCGAGCAGC AGTGGCACGT 500
FukuhonokaNIL  TGGTGGACGC GGAGTACCTG GCCAAGTACG AGAGGGCCGT GGCGCTCATG AAGCAGCTGC CCGCCGATGA CCCGCGCAGC TTCGAGCAGC AGTGGCACGT 500
KT-012         TGGTGGACGC GGAGTACCTG GCCAAGTACG AGAGGGCCGT GGCGCTCATG AAGCAGCTGC CCGCCGATGA CCCGCGCAGC TTCGAGCAGC AGTGGCACGT 500
KT-013         TGGTGGACGC GGAGTACCTG GCCAAGTACG AGAGGGCCGT GGCGCTCATG AAGCAGCTGC CCGCCGATGA CCCGCGCAGC TTCGAGCAGC AGTGGCACGT 500
                                     520                   540                   560                   580                   600
                                       |                     |                     |                     |                     |
Ppo-D1d        GCACTGCGCC TACTGCGACG CCGCCTTCGA CCAGGTCGGG TTCCCGGACC TGGAGATCCA GGTGCACAAC TGCTGGCTCT TCTTCCCATG GCACAGGTTC 600
FukuhonokaNIL  GCACTGCGCC TACTGCGACG CCGCCTTCGA CCAGGTCGGG TTCCCGGACC TGGAGATCCA GGTGCACAAC TGCTGGCTCT TCTTCCCATG GCACAGGTTC 600
KT-012         GCACTGCGCC TACTGCGACG CCGCCTTCGA CCAGGTCGGG TTCCCGGACC TGGAGATCCA GGTGCACAAC TGCTGGCTCT TCTTCCCATG GCACAGGTTC 600
KT-013         GCACTGCGCC TACTGCGACG CCGCCTTCGA CCAGGTCGGG TTCCCGGACC TGGAGATCCA GGTGCACAAC TGCTGGCTCT TCTTCCCATG GCACAGGTTC 600
                                     620                   640                   660                   680                   700
                                       |                     |                     |                     |                     |
Ppo-D1d        GTATGGTCAA TGGGTTATGG GTGCGACGAC TTGCACCTTT CTGTGCTGAA CCTCAAGGAG CCGTCACTTG TCCCTGCGTG CGTTTGCTGA ACGTGCAGGT 700
FukuhonokaNIL  GTATGGTCAA TGGGTTATGG GTGCGACGAC TTGCACCTTT CTGTGCTGAA CCTCAAGGAG CCGTCACTTG TCCCTGCGTG CGTTTGCTGA ACGTGCAGGT 700
KT-012         GTATGGTCAA TGGGTTATGG GTGCGACGAC TTGCACCTTT CTGTGCTGAA CCTCAAGGAG CCGTCACTTG TCCCTGCGTG CGTTTGCTGA ACGTGCAGGT 700
KT-013         GTATGGTCAA TGGGTTATGG GTGCGACGAC TTGCACCTTT CTGTGCTGAA CCTCAAGGAG CCGTCACTTG TCCCTGCGTG CGTTTGCTGA ACGTGCAGGT 700
                                     720                   740                   760                   780                   800
                                       |                     |                     |                     |                     |
Ppo-D1d        TCTACGTCTA CTTCCACGAG AGGATCCTCG GCAAGCTCAT CGGCGACGAC ACCTTCGCGC TGCCCTTCTG GAACTGGGAC GCGCCGGCCG GCATGACGCT 800
FukuhonokaNIL  TCTACGTCTA CTTCCACGAG AGGATCCTCG GCAAGCTCAT CGGCGACGAC ACCTTCGCGC TGCCCTTCTG GAACTGGGAC GCGCCGGCCG GCATGACGCT 800
KT-012         TCTACGTCTA CTTCCACGAG AGGATCCTCG GCAAGCTCAT CGGCGACGAC ACCTTCGCGC TGCCCTTCTG GAACTGGGAC GCGCCGGCCG GCATGACGCT 800
KT-013         TCTACGTCTA CTTCCACGAG AGGATCCTCG GCAAGCTCAT CGGCGACGAC ACCTTCGCGC TGCCCTTCTG GAACTGGGAC GCGCCGGCCG GCATGACGCT 800
                                     820                   840                   860                   880                   900
                                       |                     |                     |                     |                     |
Ppo-D1d        GCCGGCGATC TACGCCAACA GGTCGTCGCC GCTCTACGAC GAGAGGCGCG ACCCCGCCCA CCAGCCGCCG GTGCTGATCG ACCTTGACTC CAGTGGGACC 900
FukuhonokaNIL  GCCGGCGATC TACGCCAACA GGTCGTCGCC GCTCTACGAC GAGAGGCGCG ACCCCGCCCA CCAGCCGCCG GTGCTGATCG ACCTTGACTC CAGTGGGACC 900
KT-012         GCCGGCGATC TACGCCAACA GGTCGTCGCC GCTCTACGAC GAGAGGCGCG ACCCCGCCCA CCAGCCGCCG GTGCTGATCG ACCTTGACTC CAGTGGGACC 900
KT-013         GCCGGCGATC TACGCCAACA GGTCGTCGCC GCTCTACGAC GAGAGGCGCG ACCCCGCCCA CCAGCCGCCG GTGCTGATCG ACCTTGACTC CAGTGGGACC 900
                                     920                   940                   960                   980                 1,000
                                       |                     |                     |                     |                     |
Ppo-D1d        GACGCCAATA TCCCAAGAGA CCAGCAGATC GATCAGAACC TCAAGATCAT GTACCGCCAG GCCAGTAGTA CTAATTAACA ACCTCAAGAA TCCCTAAACA 1000
FukuhonokaNIL  GACGCCAATA TCCCAAGAGA CCAGCAGATC GATCAGAACC TCAAGATCAT GTACCGCCAG GCCAGTAGTA CTAATTAACA ACCTCAAGAA TCCCTAAACA 1000
KT-012         GACGCCAATA TCCCAAGAGA CCAGCAGATC GATCAGAACC TCAAGATCAT GTACCGCCAG GCCAGTAGTA CTAATTAACA ACCTCAAGAA TCCCTAAACA 1000
KT-013         GACGCCAATA TCCCAAGAGA CCAGCAGATC GATCAGAACC TCAAGATCAT GTACCGCCAG GCCAGTAGTA CTAATTAACA ACCTCAAGAA TCCCTAAACA 1000
                                   1,020                 1,040                 1,060                 1,080                 1,100
                                       |                     |                     |                     |                     |
Ppo-D1d        AAATGAGTAG CAACTTCAAA AATATTGTTA AGGTAACCAC AGAACCACTG GTCATGAAAT AACACAAATG TACGTACGCA CCAGATGATT TCGGACGCGA 1100
FukuhonokaNIL  AAATGAGTAG CAACTTCAAA AATATTGTTA AGGTAACCAC AGAACCACTG GTCATGAAAT AACACAAATG TACGTACGCA CCAGATGATT TCGGACGCGA 1100
KT-012         AAATGAGTAG CAACTTCAAA AATATTGTTA AGGTAACCAC AGAACCACTG GTCATGAAAT AACACAAATG TACGTACGCA CCAGATGATT TCGGACGCGA 1100
KT-013         AAATGAGTAG CAACTTCAAA AATATTGTTA AGGTAACCAC AGAACCACTG GTCATGAAAT AACACAAATG TACGTACGCA CCAGATGATT TCGGACGCGA 1100
                                   1,120                 1,140                 1,160                 1,180                 1,200
                                       |                     |                     |                     |                     |
Ppo-D1d        AGAAGACGCT GCTGTTCCTG GGACAGCCGT ACCGCGCCGG CGACCAGCCG GACCCGGGCG CGGGCTCCCT GGAGAACGTG CCGCACGGCA CGGTCCACGT 1200
FukuhonokaNIL  AGAAGACGCT GCTGTTCCTG GGACAGCCGT ACCGCGCCGG CGACCAGCCG GACCCGGGCG CGGGCTCCCT GGAGAACGTG CCGCACGGCA CGGTCCACGT 1200
KT-012         AGAAGACGCT GCTGTTCCTG GGACAGCCGT ACCGCGCCGG CGACCAGCCG GACCCGGGCG CGGGCTCCCT GGAGAACGTG CCGCACGGCA CGGTCCACGT 1200
KT-013         AGAAGACGCT GCTGTTCCTG GGACAGCCGT ACCGCGCCGG CGACCAGCCG GACCCGGGCG CGGGCTCCCT GGAGAACGTG CCGCACGGCA CGGTCCACGT 1200
                                   1,220                 1,240                 1,260                 1,280                 1,300
                                       |                     |                     |                     |                     |
Ppo-D1d        CTGGACTGGC GACCCGGCGC AGCCGAACTT GGAGGACATG GGCAACTTCT TCTCGGCGGC GCGCGACCCC ATCTTCTTCG CGCACCACGG CAACATCGAC 1300
FukuhonokaNIL  CTGGACTGGC GACCCGGCGC AGCCGAACTT GGAGGACATG GGCAACTTCT TCTCGGCGGC GCGCGACCCC ATCTTCTTCG CGCACCACGG CAACATCGAC 1300
KT-012         CTGGACTGGC GACCCGGCGC AGCCGAACTT GGAGGACATG GGCAACTTCT TCTCGGCGGC GCGCGACCCC ATCTTCTTCG CGCACCACGG CAACATCGAC 1300
KT-013         CTGGACTGGC GACCCGGCGC AGCCGAACTT GGAGGACATG GGCAACTTCT TCTCGGCGGC GCGCGACCCC ATCTTCTTCG CGCACCACGG CAACATCGAC 1300
                                   1,320                 1,340                 1,360                 1,380                 1,400
                                       |                     |                     |                     |                     |
Ppo-D1d        CGCCTGTGGC ACGTCTGGCG CCGCCTCCGC CCGAGCAACA CCGACTTCAC CGACCCCGAC TGGCTCGACG CCGCCTTCCT CTTCTACGAC GAGGAGGCCC 1400
FukuhonokaNIL  CGCCTGTGGC ACGTCTGGCG CCGCCTCCGC CCGAGCAACA CCGACTTCAC CGACCCCGAC TGGCTCGACG CCGCCTTCCT CTTCTACGAC GAGGAGGCCC 1400
KT-012         CGCCTGTGGC ACGTCTGGCG CCGCCTCCGC CCGAGCAACA CCGACTTCAC CGACCCCGAC TGGCTCGACG CCGCCTTCCT CTTCTACGAC GAGGAGGCCC 1400
KT-013         CGCCTGTGGC ACGTCTGGCG CCGCCTCCGC CCGAGCAACA CCGACTTCAC CGACCCCGAC TGGCTCGACG CCGCCTTCCT CTTCTACGAC GAGGAGGCCC 1400
                                   1,420                 1,440                 1,460                 1,480                 1,500
                                       |                     |                     |                     |                     |
Ppo-D1d        GCCCCGTGCG CGTGCGCGTC CGGGACTGCC TCGACCCGGC CGCGCTGCGG TACACGTACC AGGACGTCGG CCTGCCGTGG CTCAACGCCA GGCCGCCCAA 1500
FukuhonokaNIL  GCCCCGTGCG CGTGCGCGTC CGGGACTGCC TCGACCCGGC CGCGCTGCGG TACACGTACC AGGACGTCGG CCTGCCGTGG CTCAACGCCA GGCCGCCCAA 1500
KT-012         GCCCCGTGCG CGTGCGCGTC CGGGACTGCC TCGACCCGGC CGCGCTGCGG TACACGTACC AGGACGTCGG CCTGCCGTGG CTCAACGCCA GGCCGCCCAA 1500
KT-013         GCCCCGTGCG CGTGCGCGTC CGGGACTGCC TCGACCCGGC CGCGCTGCGG TACACGTACC AGGACGTCGG CCTGCCGTGG CTCAACGCCA GGCCGCCCAA 1500
                                   1,520                 1,540                 1,560                 1,580                 1,600
                                       |                     |                     |                     |                     |
Ppo-D1d        GGCGTCCGGC GGGACGCCGG CGCCCGCCAC AACCGGGAGA AGGAGGAGGA GGAGGAGGTG CTGGTCGTGG AGGGGATCGA GATCGCCGAC CATTTCAACA 1600
FukuhonokaNIL  GGCGTCCGGC GGGACGCCGG CGCCCGCCAC AACCGGGAGA AGGAGGAGGA GGAGGAGGTG CTGGTCGTGG AGGGGATCGA GATCGCCGAC CATTTCAACA 1600
KT-012         GGCGTCCGGC GGGACGCCGG CGCCCGCCAC AACCGGGAGA AGGAGGAGGA GGAGGAGGTG CTGGTCGTGG AGGGGATCGA GATCGCCGAC CATTTCAACA 1600
KT-013         GGCGTCCGGC GGGACGCCGG CGCCCGCCAC AACCGGGAGA AGGAGGAGGA GGAGGAGGTG CTGGTCGTGG AGGGGATCGA GATCGCCGAC CATTTCAACA 1600
                                   1,620                 1,640                 1,660                 1,680                 1,700
                                       |                     |                     |                     |                     |
Ppo-D1d        AGTTCGTCAA GTTCGACGTG TTGGTGAACG AGCCCGAGGG CGGAGTGGGC AGCACGCCGG CGACGGCGAC GGGGTACTGC GCTGGGAGCT TCGCGCATAC 1700
FukuhonokaNIL  AGTTCGTCAA GTTCGACGTG TTGGTGAACG AGCCCGAGGG CGGAGTGGGC AGCACGCCGG CGACGGCGAC GGGGTACTGC GCTGGGAGCT TCGCGCATAC 1700
KT-012         AGTTCGTCAA GTTCGACGTG TTGGTGAACG AGCCCGAGGG CGGAGTGGGC AGCACGCCGG CGACGGCGAC GGGGTACTGC GCTGGGAGCT TCGCGCATAC 1700
KT-013         AGTTCGTCAA GTTCGACGTG TTGGTGAACG AGCCCGAGGG CGGAGTGGGC AGCACGCCGG CGACGGCGAC GGGGTACTGC GCTGGGAGCT TCGCGCATAC 1700
                                   1,720                 1,740                 1,760                 1,780                 1,800
                                       |                     |                     |                     |                     |
Ppo-D1d        GCCGCACATG GTCCGGCCCG AGGAGATGAG GAAGGGACCG GTCAAGACGG TGGCGAGGTT CGGCGTGTGC GACCTGATGG ACGACATCGG GGCGGACGAC 1800
FukuhonokaNIL  GCCGCACATG GTCCGGCCCG AGGAGATGAG GAAGGGACCG GTCAAGACGG TGGCGAGGTT CGGCGTGTGC GACCTGATGG ACGACATCGG GGCGGACGAC 1800
KT-012         GCCGCACATG GTCCGGCCCG AGGAGATGAG GAAGGGACCG GTCAAGACGG TGGCGAGGTT CGGCGTGTGC GACCTGATGG ACGACATCGG GGCGGACGAC 1800
KT-013         GCCGCACATG GTCCGGCCCG AGGAGATGAG GAAGGGACCG GTCAAGACGG TGGCGAGGTT CGGCGTGTGC GACCTGATGG ACGACATCGG GGCGGACGAC 1800
```

```
                  1,820                 1,840                 1,860                 1,880
                      |                     |                     |                     |
Ppo-D1d       GACCAGACGG TGGTGGTGTC GCTCGTACCC AGGTGCGGCG GTGAGCTGGT CACCGTTGGC GGCGTCAGCA TCAGCTACCT CAAGTGA 1887
FukuhonokaNIL GACCAGACGG TGGTGGTGTC GCTCGTACCC AGGTGCGGCG GTGAGCTGGT CACCGTTGGC GGCGTCAGCA TCAGCTACCT CAAGTGA 1887
KT-012        GACCAGACGG TGGTGGTGTC GCTCGTACCC AGGTGCGGCG GTGAGCTGGT CACCGTTGGC GGCGTCAGCA TCAGCTACCT CAAGTGA 1887
KT-013        GACCAGACGG TGGTGGTGTC GCTCGTACCC AGGTGCGGCG GTGAGCTGGT CACCGTTGGC GGCGTCAGCA TCAGCTACCT CAAGTGA 1887
```

Supplemental Fig. 5. Aliment of the ORF sequences of the *ppo-D1d-like* alleles. The *ppo-D1d* sequence is from GenBank accession no. EU371657. The sequence of 'Fukuhonoka-NIL' (GenBank accession no. LC843431) is a consensus sequence generated from NGS data. The sequence of KT120-012 (GenBank accession no. LC843432) and KT120-013 (GenBank accession no. LC843433) were obtained by direct sequencing.

```
Start codon
Chinese Spring  ATGGAGAGCA CTCGCGTGCT ACCGAGTGCC AACCATCGCA TGCCATGCAG CCTCCAAACC TTTCCGCGAC GCAACGTTCT CCGTGCCCTT CACCGCCGCA AGGACGCAAG 110
AL8/78          ATGGAGAGCA CTCGCGTGCT ACCGAGTGCC AACCATCGCA TGCCATGCAG CCTCCAAACC TTTCCGCGAC GCAACGTTCT CCGTGCCCTT CACCGCCGCA AGGACGCAAG 110

Chinese Spring  GCAGCCACGT CGTATCTCAA TCTCATGCGA GGCGACCGGC GGCGGTCGCG TCGACCGCCG TGAGGTGCTC CTCGGCCTCG GCGGCGCCGC AGCTGCGGGG CTGGCCACGG 220
AL8/78          GCAGCCACGT CGTATCTCAA TCTCATGCGA GGCGACCGGC GGCGGTCGCG TCGACCGCCG TGAGGTGCTC CTCGGCCTCG GCGGCGCCGC AGCTGCGGGG CTGGCCACGG 220

Chinese Spring  ACCAAGGTCG AGGCGCGATC GCCGCGCCCA TCCAGGCCCC GGACCTCCGC AACTGCCAAA CGCCCGACCT CCCGAACACG CCGCCGGACA CCAACTGCTG CCCGACGCCC 330
AL8/78          ACCAAGGTCG AGGAGCGATC GCCGCGCCCA TCCAGGCCCC GGACCTCCGC AACTGCCAAA CGCCCGACCT CCCGAACACG CCGCCGGACA CCAACTGCTG CCCGACGCCC 330

Chinese Spring  GGCACCGGCA TCACCGACTT TGAGCTGCCG CCCGCTTCCT CGCCGCTCCG CGTGCGTCCG GCAGCGCACC TGGTGGACGC GGAGTACCTG GCCAAGTACG AGAGGGCCGT 440
AL8/78          GGCACCGGCA TCACCGACTT TGAGCTGCCG CCCGCTTCCT CGCCGCTCCG CGTGCGTCCG GCAGCGCACC TGGTGGACGC GGAGTACCTG GCCAAGTACG AGAGGGCCGT 440

Chinese Spring  GGCGCTCATG AAGCAGCTGC CTGCCGATGA CCCGCGCAGC TTCGAGCAGC AGTGGCACGT GCACTGCGCC TACTGCGACG CCGCCTTCGA CCAGGTCGGG TTCCCGGACC 550
AL8/78          GGCGCTCATG AAGCAGCTGC CCGCCGATGA CCCGCGCAGC TTCGAGCAGC AGTGGCACGT GCACTGCGCC TACTGCGACG CCGCCTTCGA CCAGGTCGGG TTCCCGGACC 550

Chinese Spring  TTGAGATCCA GGTGCACAAC TGCTGGCTCT TCTTCCCATG GCACAGGTTC GTATGGTCAA TGGGTTAATG GGTGCGACGA CTTGCACCTT TCTGTGCTGA ACCTCAAGGA 660
AL8/78          TGGAGATCCA GGTGCACAAC TGCTGGCTCT TCTTCCCATG GCACAGGTTC GTATGGTCAA TGGGTTA-TG GGTGCGACGA CTTGCACCTT TCTGTGCTGA ACCTCAAGGA 659

Chinese Spring  GCCGTCACTT GTCCCTGCGT GCGTTTGCTG AACGTGCAGG TTCTACGTCT ACTTCCACGA GAGGATCCTC GGCAAGCTCA TCGGCGACGA CACCTTCGCG CTGCCCTTCT 770
AL8/78          GCCGTCACTT GTCCCTGCGT GCGTTTGCTG AACGTGCAGG TTCTACGTCT ACTTCCACGA GAGGATCCTC GGCAAGCTCA TCGGCGACGA CACCTTCGCG CTGCCCTTCT 769

Chinese Spring  GGAACTGGGA CGCGCCGGCC GGCATGACGC TGCCGGCGAT CTACGCCAAC AGGTCGTCGC CGCTCTACGA CGAGAGGCGC GACCCCGCCC ACCAGCCGCC GGTGCTGACC 880
AL8/78          GGAACTGGGA CGCGCCGGCC GGGATGACGC TGCCGGCGAT CTACGCCAAC AGGTCGTCGC CGCTCTACGA CGAGAGGCGC GACCCCGCCC ACCAGCCGCC GGTGCTGATC 879

Chinese Spring  GACCTTGACT CCAGTGGGAC CGACGCCAAT ATCCCAAGAG ACCAGCAGAT CGATCAGAAC CTCAAGATCA TGTACCGCCA GGCCAGTAGT ACTAATTAAC AACCTCAAGA 990
AL8/78          GACCTTGACT CCAGTGGGAC CGACGCCAAT ATCCCAAGAG ACCAGCAGAT CGATCAGAAC CTCAAGATCA TGTACCGCCA GGCCAGTAGT ACTAATTAAC AACCTCAAGA 989

Chinese Spring  ATCCCTAAAC AAAATGAGTA GCAACTTCAA AAATATTGTT AA----GGTA ACCACAGAAC CACTGGTCAT GAAATAACAC AAATGTACGT ACGCACCAGA TGATTTCGGA 1096
AL8/78          ATCCCTAAAC AAAATGAGTA GCAACTTCAA AAATATTGTT AATTAAGGTA ACCACAGAAC CACTGGTCAT GAGATAACAC AAATGTACGT ACGCACCAGA TGATTTCGGA 1099

Chinese Spring  CGCGAAGAAG ACGCTGCTGT TCCTGGGACA GCCGTACCGC GCCGGCGACC AGCCGGACCC GGGCGCGGGC TCCCTGGAGA ACGTGCCGCA CGGCACGGTC CACGTCTGGA 1206
AL8/78          CGCGAAGAAG ACGCTGCTGT TCCTGGGACA GCCGTACCGC GCCGGCGACC AGCCGGACCC GGGCGCGGGC TCCCTGGAGA ACGTGCCGCA CGGCACGGTC CACGTCTGGA 1209

Chinese Spring  CTGGCGACCC GGCGCAGCCG AACTTGGAGG ACATGGGCAA CTTCTTCTCG GCGGCGCGCG ACCCCATCTT CTTCGCGCAC CACGGCAACA TCGACCGCCT GTGGCACGTC 1316
AL8/78          CTGGCGACCC GGCGCAGCCG AACTTGGAGG ACATGGGCAA CTTCTTCTCG GCGGCGCGCG ACCCCATCTT CTTCGCGCAC CACGGCAACA TCGACCGCCT GTGGCACGTC 1319

Chinese Spring  TGGCGCCGCC TCCGCCCGAG CAACACCGAC TTCACCGACC CCGACTGGCT CGACGCCGCC TTCCTCTTCT ACGACGAGGA GGCCCGCCCC GTGCGCGTGC GCGTCCGGGA 1426
AL8/78          TGGCGCCGCC TCCGCCCGAG CAACACCGAC TTCACCGACC CCGACTGGCT CGACGCCGCC TTCCTCTTCT ACGACGAGGA GGCCCGCCCC GTGCGCGTGC GCGTCCGGGA 1429

Chinese Spring  CTGCCTCGAC CCGGCCGCGC TGCGGTACAC GTACCAGGAC GTCGGCCTGC CGTGGCTCAA CGCCAGGCCG GCCAAGGCGT CCGGCGGG-- ACGCCGGCGC CCGCCACAAC 1534
AL8/78          CTGCCTCGAC CCGGCCGCAC TGCGGTACAC GTACCAGGAC GTCGGCCTGC CGTGGCTCAA CGCCAGGCCG GCCAAGGCGT CCGGCGGGGG ACGCCGGCGC CCGCCACAAC 1539

Chinese Spring  CGGTACCCTC CCTGCCACCC TGGACAGGAC CATACGGGTG ACGGTGACGA GGCCCAGAGT GTCCAGGAGC CGCCGGGAGA AGGAGGAGGA GGAGGAGGTG CTGGTCGTGG 1644
AL8/78          CGGTACCCTC CCTGCCACCC TGGACAGGAC CATACGGGTG ACGGTGACAA GGCCCAGAGT GTCCAGGAGC CGCCGGGAGA AGGAGGAGGA GGAGGAGGTG CTGGTCGTGG 1649

                             →Forward primer STS01F
Chinese Spring  AGGGGATCGA GATCGCCGAC CATTTCAACA AGTTCGTCAA GTTCGACGTG TTGGTGAACG AGCCCGAGGG CGGAGTGGGC AGCACGCCGG CGACGGCGAC GGGGTACTGC 1754
AL8/78          AGGGGATCGA GATCGCCGAC CATTTCAACA AGTTCGTCAA GTTCGACGTG TTGGTGAACG AGCCCGAGGG CGGAGTGGGC AGCACGCCGG CGACGGCGAC GGGGTACTGC 1759

Chinese Spring  GCTGGGAGCT TCGCGCATAC GCCGCACATG GTCCGGCCCG AGGAGATGAG GAAGGGACCG GTCAAGACGG TGGCGAGGTT CGGCGTGTGC GACCTGATGG ACGACATCGG 1864
AL8/78          GCTGGGAGCT TCGCGCATAC GCCGCACATG GTCCGGCCCG AGGAGATGAG GAAGGGACCG GTCAAGACGG TGGCGAGGTT CGGCGTGTGC GACCTGATGG ACGACATCGG 1869

                                                                                                                stop codon
Chinese Spring  GGCGGACGAC GACCAGACGG TGGTGGTGTC GCTCGTACCC AGGTGCGGCG GTGAGCTGGT CACCGTTGGC GGCGTCAGCA TCAGCTACCT CAAGTGAAGT TACCTAATGT 1974
AL8/78          GGCGGACGAC GACCAGACGG TGGTGGTGTC GCTCGTACCC AGGTGCGGCG GTGAGCTGGT CACCGTTGGC GGCGTCAGCA TCAGCTACCT CAAGTGAAGT TACCTAATGT 1979

                                                                                     →3 kb insertion
Chinese Spring  GGTCCGCTCT CGCGTCGGTG GCTCGCCGTG GTATAGAATC TATGTAATGG TGATGGTGAT GAATTATAAA TAGGGGTCTG CTAATTCTCA GTCGACTGAG AATTAGTGAA 2084
AL8/78          GGTCCGCTCT CGCGTCGGTG GCTCGCCGTG GTATAGAATC TATGTAATGG TGATGGTGAT GAATTATAAA T--------- ---------- ---------- ---------- 2050

Chinese Spring  ATCTCAGTCG ACAGGATTAA CCATACGATT CGATTGACCT GTATTTATCG CTGATGTATT TTTGTTTTTC AAAACCTATT GGGCTTTCTC TATTTGAGCG GCTGGCGGCT 2194
AL8/78          ---------- ---------- ---------- ---------- ---------- ---------- ---------- ---------- ---------- ---------- ---------- 2050

                           Reverse primer STS01R←
Chinese Spring  GCATGGGCTG TGGGCTACGG CTTGTGGTCC TTCTCATTTT CCCTTTTCTG TTTTTTTTAT TTTCTTAATA GATATCCCAC GTAAATATAT TGTATTTTTT GTTTTTCTGT 2304
AL8/78          ---------- ---------- ---------- ---------- ---------- ---------- ---------- ---------- ---------- ---------- ---------- 2050

Chinese Spring  TAGGTTTGTG TGTATACAAA GAAGATACGT GTACTTATCT AGCTTTTTTT CTACTTTATT ATTTATTTTC TATTTTCTCA TTTTGTGTAT TTATTTTTAT ATGTTTTATT 2414
AL8/78          ---------- ---------- ---------- ---------- ---------- ---------- ---------- ---------- ---------- ---------- ---------- 2050

Chinese Spring  TGTCTGTTTT ACTTCTATTT CTACTTTTGC CTATTTTTAT CATTCATCTT TGGAAGATTT AAGTTTTGTA GAATTTACAT TTTGACACGC ATGGACAATT TTTATTCTCA 2524
AL8/78          ---------- ---------- ---------- ---------- ---------- ---------- ---------- ---------- ---------- ---------- ---------- 2050

Chinese Spring  CTTGTTGTTG TCTTTTCTAC ATGTTTTTGT TTTAATTTTA TTCATGTAGT ACACAAAAAT ACATTTTTCA TCATGTTTAT ATGTTTTATT TGTTTGTTTT TACTTCTATT 2634
AL8/78          ---------- ---------- ---------- ---------- ---------- ---------- ---------- ---------- ---------- ---------- ---------- 2050

Chinese Spring  TCTTCTTCTT TGTTTGTTTG TTTTTGGGCG TGATTCAAAG GGGAACGGGC AGATATGTGT GCTCCCGTCG CAATACCGAT GGAGATGGGT GACGCAATAC CGATGGAGAT 2744
AL8/78          ---------- ---------- ---------- ---------- ---------- ---------- ---------- ---------- ---------- ---------- ---------- 2050

Chinese Spring  GGGTGACGCT GCTGATGGAG TATTTTATCT CCGTTATTAT CCGTTGTCTT TTTTGTCTTT TTTCTTTTTT GCTTCTGGTT CGAGTAGGTT GAATCAAGTA CAGATCATTA 2854
AL8/78          ---------- ---------- ---------- ---------- ---------- ---------- ---------- ---------- ---------- ---------- ---------- 2050

Chinese Spring  GGGGTACCAG AAGGTTAAAT TAAGCAGAGA TTAGAAGTAA TCTACTTTCT TTTTTGTTTC TGGTTCGAGT AGGTTGAATC AAGTACAAAT CAGTAGAGGT ACTAGAAGGT 2964
AL8/78          ---------- ---------- ---------- ---------- ---------- ---------- ---------- ---------- ---------- ---------- ---------- 2050

Chinese Spring  TGAATTAAGT AGAGATTAGT AGTAATCTAC TTTCTTTTTT GTTTCTGGTT CAAGTAGGTT CAATCAAGTA CAAATCAGTA GGGTACCAGA AGGTTGAATT AAGTACAGAA 3074
AL8/78          ---------- ---------- ---------- ---------- ---------- ---------- ---------- ---------- ---------- ---------- ---------- 2050

Chinese Spring  TCAGTAGGTT GAATCAATTG CATGTCTTCT AGCCCGACCG TAACTAGACT TTTTTGTTTT GTTGGATTGG GCACAGGTAG GAGGGGGCGG GCAAGTTGCA AGCCAGACAA 3184
AL8/78          ---------- ---------- ---------- ---------- ---------- ---------- ---------- ---------- ---------- ---------- ---------- 2050

Chinese Spring  CAGCCTCACA ACTGCAAGTG TCGTACCTGA CCGCAACTTG CATGTCCCCC AACATGGTTG CAACTGGACT TTTTTGTTTT GTTGGAGGGG GCACCGGTAG AGGGGCGGGC 3294
AL8/78          ---------- ---------- ---------- ---------- ---------- ---------- ---------- ---------- ---------- ---------- ---------- 2050

Chinese Spring  AAGTTGTAAG CCAGGCAACA GCCTCACATG CTGCAAGTGC TGCACCTGAC CGCAACTTGC ATGTCCCTCA ACATGGTTGC AACTTGACTT TTATTTTGTC GGAGGGGGTG 3404
AL8/78          ---------- ---------- ---------- ---------- ---------- ---------- ---------- ---------- ---------- ---------- ---------- 2050
```

```
               3,420      3,440      3,460      3,480      3,500      3,520
Chinese Spring GCGGGAGAGG GGGCGGGCCA GTTGCATGTC CGACACTAGG CATGCAACTG CAAGTGTCAC CCCCGACCGC AACCTCATCT CTTCTAACCC CGATTGCAAT TGAACTTTTT 3514
AL8/78         ---------- ---------- ---------- ---------- ---------- ---------- ---------- ---------- ---------- ---------- ---------- 2050
               3,540      3,560      3,580      3,600      3,620
Chinese Spring TGTTCTGCCA AAGGGGGCGT CGGGAGAGGG GGCAGGAAAG TTGCAAGCCC GGCAACAGCC CCGCAATAGC AATGTCACAC CCGACCGCAA CTGCATGTCT TCTAACCCGA 3624
AL8/78         ---------- ---------- ---------- ---------- ---------- ---------- ---------- ---------- ---------- ---------- ---------- 2050
               3,640      3,660      3,680      3,700      3,720      3,740
Chinese Spring CCGCAACTTG AATTTTTGTG TTCTGTTGGA GGGGGCGCCG GGAGAGGGGG CGGGCCAGTT GCAAGCCCGA CAACAACCCC GCAACTTCAT GTCCCCCAAC ATGATTGCAA 3734
AL8/78         ---------- ---------- ---------- ---------- ---------- ---------- ---------- ---------- ---------- ---------- ---------- 2050
               3,760      3,780      3,800      3,820      3,840
Chinese Spring CAACCCCGCA ACTTTTGTTA AGGGGGTGGC GGGAGAGGGA GAGGGCCGGG CCAGTTGCAA GCCCGACAAT AGCCCCGCAA CTGCAAGTGC CGCACCAGAC CACAACTGCT 3844
AL8/78         ---------- ---------- ---------- ---------- ---------- ---------- ---------- ---------- ---------- ---------- ---------- 2050
               3,860      3,880      3,900      3,920      3,940      3,960
Chinese Spring TGTCCCCCAA CATGGTTGCA ACTCGACTTT TGTTTTGTTG AAGGGGGCGC CTGGAGAGGG GGCGGGCCAG TTGCAAGCCC AACAACTGCC CCACAACTGC AAGTGTCGAA 3954
AL8/78         ---------- ---------- ---------- ---------- ---------- ---------- ---------- ---------- ---------- ---------- ---------- 2050
               3,980      4,000      4,020      4,040      4,060
Chinese Spring CCCGACCGCA CCTGCATGTC CCCCAACATG GTTGCAACTC GACTTTTGTT TTGTCGGAGG GGGCGTCGAG AGAGGGGGCG GGCCAGTTGC AAGCCCGACA ACAGCCCTGC 4064
AL8/78         ---------- ---------- ---------- ---------- ---------- ---------- ---------- ---------- ---------- ---------- ---------- 2050
               4,080      4,100      4,120      4,140      4,160      4,180
Chinese Spring AACTGCAAGT GCCGCACCCG ACCGCAACTG GACTTTTTTG TTCTATCAGA GGGGGCGGGG GGGGGGGAGG CAGTTGCAAG CCCGACAACA ACCCCACAAC TGCATGTCCC 4174
AL8/78         ---------- ---------- ---------- ---------- ---------- ---------- ---------- ---------- ---------- ---------- ---------- 2050
               4,200      4,220      4,240      4,260      4,280
Chinese Spring CATGGTTGCA ACTCGACTTT TGTTTTGTCG GAGGGGGCAC CGGGAGAGGG GCGGGCTAGT TGCAAGCCCG AAAACAGCAG TGCAACTGCA AGTGCCGCAC CCGATAGCAA 4284
AL8/78         ---------- ---------- ---------- ---------- ---------- ---------- ---------- ---------- ---------- ---------- ---------- 2050
               4,300      4,320      4,340      4,360      4,380      4,400
Chinese Spring ATGCATGCCC CCAACATGGT TGCAACTCGA TTTTTGTTTT GTCGGAGGGG GCACCGGGAG AGGGGGCGGG CTAGTTGCAA GCCCGACAAC AGCCCCGTAA ATGCAAGTGC 4394
AL8/78         ---------- ---------- ---------- ---------- ---------- ---------- ---------- ---------- ---------- ---------- ---------- 2050
               4,420      4,440      4,460      4,480      4,500
Chinese Spring CGCACACGAC CGCAACTACA TGTCCCCCAA CATGGTTGCA ACTCGACTTT TGTTTTGTCA GAGTGGGCGC CGGGAGAGAG GGAGGGCTAG TTGCAAGCCC GACAACAGCC 4504
AL8/78         ---------- ---------- ---------- ---------- ---------- ---------- ---------- ---------- ---------- ---------- ---------- 2050
               4,520      4,540      4,560      4,580      4,600      4,620
Chinese Spring CCGCAACTGC AAGTGCCGCA CCCGACCGCA AGTGCATGTC CCCCAACATG GTTGTAACTC GACTTTTGTT TTGTCAGAGT GGGCGCCGGG AGATGGGGCG GGCCACTTGC 4614
AL8/78         ---------- ---------- ---------- ---------- ---------- ---------- ---------- ---------- ---------- ---------- ---------- 2050
               4,640      4,660      4,680      4,700      4,720
Chinese Spring AAGCCCGACA ACAGCCCCAC AACTGCAAAT GTCGCACCCG ACCGCAACTG CATGTTTTAA CCCGATCGCA ATTGGACTTT TTTCTGTGGG AGGGTGCGCC GGGAGAGGAG 4724
AL8/78         ---------- ---------- ---------- ---------- ---------- ---------- ---------- ---------- ---------- ---------- ---------- 2050
               4,740      4,760      4,780      4,800      4,820      4,840
Chinese Spring GCGGGCGAGT GAAATGAAAC GTGTACTCCT AGTGACACAC ATACGTACAT ATGTGACAAG ATGACACACG TGTAGACATG CTAATCACGT ATAGTACACT CTACAGACCC 4834
AL8/78         ---------- ---------- ---------- ---------- ---------- ---------- ---------- ---------- ---------- ---------- ---------- 2050
               4,860      4,880      4,900      4,920      4,940
Chinese Spring ATATCCATAC ATCTGTACGT GGGCATATGT ACGGGTGGCA CCGTACGTCC GTGATCTCCG AAGAAAAAGA AAGAAAAGGG TCCGGCCTAA TGACAACAGA AAAAATGAGC 4944
AL8/78         ---------- ---------- ---------- ---------- ---------- ---------- ---------- ---------- ---------- ---------- ---------- 2050
               4,960      4,980      5,000      5,020      5,040      5,060
Chinese Spring AGCCCAGCCC AAAATAACAA ACAAACAAAC AAGTAAAATG AAATAAGCCC AAGTGCTAAG CCGATTCAAC CCAGCAAAAA TAGAGCTAGT TAACAGGCCT AATCTATATG 5054
AL8/78         ---------- ---------- ---------- ---------- ---------- ---------- ---------- ---------- ---------- ---------- ---------- 2050
               5,080      5,100      5,120      5,140      5,160
Chinese Spring TGATGACGTT AGCCGACTGA GACTTGGCGA AGTCTCAGTC GACTGAGCCG TAGACAGACC CTTATAAATA AGCTACCGCA AGTGACTGCC GATGAAGCAA GCACCCATCA 5164
AL8/78         ---------- ---------- ---------- ---------- ---------- ---------- ---------A AGCTACCGCA AGTGACTGCC GATGAAGCAA GCACCCATCA 2091
               5,180      5,200      5,220      5,240      5,260      5,280
Chinese Spring GCTCTCTGGT CGGCGTTCAC TGTGGTATGG AATCTATGGT GCGTCTCCTC GTATTGTATT GCAGCACGTA CGTACGTGCG TATGTCCTAT GTGTAATGGT GATTCCAAAT 5274
AL8/78         GCTCTCTGGT CGGCGTTCAC TGTGGTATGG AATCTATGGT GCGTCTCCTC GTATTGTACT GCAGCACGTA CGTACGTGCG TATGTCCTAT GTGTAATGGT GATTCCAAAT 2201
               5,300      5,320      5,340      5,360      5,380
Chinese Spring AAGAACAGCT GGAGTTAATG GGACTGGTGG CTCGGTGCGT ACATCCTACC TATACTCCTG TGCGCAGCAC CATACTAGTT ACTACCTCCG TTTCAGTTTA CAAGTCCTGC 5384
AL8/78         AAGAACAGCT GGAGTTAATG GGACTGGTGG CTCGGTGCGT ACATCCTACC TATACTCCTG TGCGCAGCAC CATACTAGTT ACTACCTCCG TTTCAGTTTA CAAGTC---- 2307
               5,400      5,420      5,440      5,460      5,480      5,500
Chinese Spring GCGTATATCT AGGTTGTCAA TTTTATTACC CTAATATAAA CTATATAACA TAAAAATTAT ACCGTTTGAA AATAGAACAT CTGAAGTTTA TATTGGTATA TTTTTTGTAA 5494
AL8/78         GCGTATATCT AGATTGTCAA TTTTATCACC CTAATATAAA CTATATAGCA CAAAAATTAT ACCGTTTGAA AATAGAACAT CTGAAGTTTA TATTGGTATA TTTTTTGTAA 2417
               5,520      5,540      5,560      5,580      5,600
Chinese Spring TATATGGCTT GTATTAGATT GGTTAATTTT TTTTAAGGAT ACGTCAAGCA GACGTATCGA GATCAAAGAT AGAAGATGCT AAGATGGCGA GTTATTACAG CGTTACAACA 5604
AL8/78         TATATGGCTT GTATTAGGTT GGTTAATTTT TTTCAAGGAT ACGTCAAGCA GACGTATCGA GATCAAAGAT AGAAGATGCC AAGATGGCGA GTTATTACAG CGTTACAACA 2527
               5,620      5,640      5,660      5,680      5,700      5,720
Chinese Spring ACCAAAAACC CACGGCTGCA CGAGCCTGAG ACCGCATGCT GACTACTAGC TATCGCCCAC CCTATCTATC CTGACTGGGA GCGATCCTGC CTCCCGCAAC AGTGCCGCAG 5714
AL8/78         ACCAAAAACC CACGGCTGCA CGAGCCTGAG ACCGCATGCT GACTACTAGC TATCGCCCAC CCTATCTATC CTGACTGGGA GCGATCCTGC CTCCCGCAAC AGTGCCGTAG 2637
               5,740      5,760      5,780      5,800      5,820
Chinese Spring CCCTCCAGTT TTGTCCCTCC GCTTCGATCT GTCGCAGAAC CTCATTCACG GAAGGTGAAG CTCCGTTGAA CACGATGTCA TTCCTGTGTT TCCACAGGGT CCAAGCCACT 5824
AL8/78         CCCTCCAGTT TTGTCCCTCC GCTTCGATCT GTCGCAGAAC CTCATTCACG GAAGGTGAAG CTTCGTTGAA CATGATGTCA TTCCTGTGTT TCCACAGGGT CCAAGCCACT 2747
               5,840      5,860      5,880      5,900      5,920      5,940
Chinese Spring AGGGTGATCA CCGTCCATGA TTTTTTTCTT TTGTTCTTCT AAATGTTGAG GGAGGTCCAC CATCCCACGA GTTCAGCCTC CGGCGCAGGT GTCCAGTGAG GCTTTCCCCA 5934
AL8/78         AGGGTGATCA CCGTCCATGA TTCTTTTCTT TTGTTCTTCT GAATGTTGAG GGAGGTCCAC CATCCCACGA GTTCAGCCTC CGGCGCAGGT GTCGAGTGAG GCTTTCCCCA 2857
               5,960      5,980      6,000      6,020      6,040
Chinese Spring GTTACCGATG ATGACCGCCC AAACTTGCCG AGGGAGAACA CATCCAAGGA GCAGGTGATC AATCGTTTCT GGCTCATGGT CGCAGAACGG GAACGCCGCT GGGTGAGGCA 6044
AL8/78         GTTGCCGATG ATGACCGCCC TAACTTGCCT AGCGAGAACA CATCCAAGGA GCAGGTGATC AATCGTTTCT GGCTCATGGT CGCAGAACGG GCACGCCGCT GGGTGAGGCA 2967
               6,060      6,080      6,100      6,120      6,140      6,160
Chinese Spring GTAGACGTCT CTGTAATCTG TCCGACGTCC AACATCTTCT CTTTGCCGCT AGCCAGGAAA AAAACTTGCA AGTTGTCGGT GCTCTAGACT TCCATATTTC CGTGGCCCCT 6154
AL8/78         GTAGACGTGT CTGTAATCTG TCTGACGTCC AGCATCTTCT CTTTGCCACT AGCCAGGAAA AAAACTTGCA AGTTGTCGGT GCTCTGGACT TCCATATTTC CGTGGCCCCT 3077
               6,180      6,200      6,220      6,240      6,260
Chinese Spring GCCACCTCAA TTCTTGCGGC AAAGTGAGCA CGATAGGCTG ATCGGGCGGA GAACAGACCA TTTTCTTCCC ATGTCCACAC AATCTTGTCA TCGATCCCTT CCGCAAGTTC 6264
AL8/78         GCCACCTCAA TTCTTGCGGC AAAGTGAGCA CGATAGGCTG ATCAGGCAGA GAACAGACCA TTTTCTTCCC ATGTCCACAC AATCTTGTCA TCGATCCCTT CCGCAAGTTC 3187
               6,280      6,300      6,320      6,340      6,360      6,380
Chinese Spring GACACTCCGT AGTCGATCAA CCAATCTCAG GAACTCTTGT ATGCCCTGGG CGTCCATGTT GGGTGACACG TCACGCCACC ATTGCCCTGA CATTGCTTCT CTCACTGTTC 6374
AL8/78         GAC--TCCGT AGTCGATCAA CCAATCTCAG GAACTCTTGT ATGCCCTGGG CGTCCATGTT GGGTGACACG TCACGCCACC ATTGCCCTGA CATTGCTTCT CTCACTGTTC 3295
               6,400      6,420      6,440      6,460      6,480
Chinese Spring TGACCTTGGC TACGCTCTTC TTGACGACAC TGAATAGGTT TGGCATGATG TCATGTATGC GTCCTTCCTG GAGCCACCAA TCCGTCCAGA ATAGTACCGT GGCTCCATCC 6484
AL8/78         TGACCTTGGA TGCGCTCTTC TTGATGACAC TGAATAGGTT TGGCATGATG TCATGTATGC GTCCCTCCTG GAGCCACCAA TCCGTCCAGA ATAGTACCGT GGCTCCATCC 3405
               6,500      6,520      6,540      6,560      6,580      6,600
Chinese Spring CCCAAGATGC ACTTCGTGGC TGCTCTGTAA ATGGCTAGCG AGTCTGATGA GACTTGGAGA TTAAATTCTG CCCATGGTCT GGCCGTATCC GTCCGTGCCA GCCACGACCA 6594
AL8/78         CCCAAGATGC ACTTCGTGGC TGCTCTGTAA ATGGCTAGCG AGTCTGATGA GACTTGGAGA TTAAATTCTG CCCATGGTCT GGCCGTATCC GTCCGTGCCA GCCACGACCA 3515
               6,620      6,640      6,660      6,680      6,700
Chinese Spring TCTCGCTCTC AGCGAAGTAT TGAGTAGGCA CAAGTTGGGC ACTCCCAGCC CACCGTATTC CTTGGGCGAA CAGACTTTGT CCCAGGCCAC CAAACAGTGC CCTCCGTGCA 6704
AL8/78         TCTCGCTCTC AGCGAAGTAT TGAGTAGGCG CAAGTTGGGC ACTCCCAGCC CACCGTATTC CTTGGGCGAA CAGACTTTGT CCCAGGCCAC CAAACAGTGC CCTCCGTGCA 3625
               6,720      6,740      6,760      6,780      6,800      6,820
Chinese Spring TGTCGCGTCG ACCCTTCCAC AGAAACCCTC TACAAATTTT GTTGGCAGCT TCTATTATCT TGACTGGAAG GTCCAACGCC ATCATGGCGT GAATTGGCAT AGATGCCAGG 6814
AL8/78         TGTCGCGTCG ACCCTTCCAC AGAAACCCTC TACAAATTTT GTTGGCAGCT TCTATTATCT TGACTGGAAG GTCCAACGCC ATCATGGCGT GAATTGGCAT AGATGCCAGG 3735
```

```
                              6,840               6,860               6,880               6,900               6,920
                                  |                   |                   |                   |                   |
Chinese Spring  GTGGTCTTCA  CCAGCTCCAG  TCGTCCTGCA  CGATACAGTA  GTCTCGCTTG  CCAAGCGGGT  AGTCTGTCAG  CCATCTTATC  CACCATATAT  TGCAGTTGAC  TCGCTGTGAT 6924
        AL8/78  GTGGTCTTCA  CCAGCTCCAG  TCGTCCTGCA  CGATACAGTA  GTCTCGCTTG  CCAAGCGGGT  AGTCTGTCAG  CCATCTTATC  CACCATATAT  TGCAGTTGAC  TCGCTGTGAT 3845
                     6,940               6,960
                         |                   |
Chinese Spring  CTTCCGTAGT  CCCAACGGCA  AGCCAAGATA  TCTACA 6960
        AL8/78  CTTCCGTAGT  CCCAACGGCA  AGCCAAGATA  TCTACA 3881
```

Supplemental Fig. 6. Alignment of the sequence from the *Ppo-D1* gene to downstream of the 3'UTR region. The sequences of Chinese Spring' and ‘AL8/78' are from the Wheat Chinese Spring IWGSC RefSeq v2.1 genome assembly (https://wheat-urgi.versailles.inra.fr/Seq-Repository/Assemblies) or *Ae. tauschii* Aet v5.0 genome assembly (NCBI BioProject PRJNA341983). Start and stop codons and primer positions are shown in yellow.
