# Supplementary material for: Development and validation of a new co-dominant DNA marker for selecting the null allele of polyphenol oxidase gene Ppo-D1 in common wheat (Triticum aestivum L.)
Source: Breed Sci. 2025 Apr 4;75(2):102–10. doi: 10.1270/jsbbs.24071 (PMC12395200; doi:10.1270/jsbbs.24071)
Supplement: Supplementary file 2 — Supplemental Tables [file 75_102_s2.pdf]

doi: 10.1270/jsbbs.24071

Supplemental Table 1. Plant materials used in this study.

variety /strain name	species	country	*Ppo-A1* allele	*Ppo-D1* allele	Reference
Fukuhonoka	*Triticum aestivum* L.	Japan	*Ppo-A1b*	*Ppo-D1a*	Nakamaru *et al.* 2023, Figure 2
Fukuhonoka NIL	*Triticum aestivum* L.	Japan	*ppo-A1i*	*ppo-D1d*	Nakamaru *et al.* 2023, Figure 4
Chinese Spring	*Triticum aestivum* L.	China	*Ppo-A1b*	*Ppo-D1a*	He *et al.* 2007
Yumechikara	*Triticum aestivum* L.	Japan	*Ppo-A1a*	*Ppo-D1b*	Kobayashi *et al.* 2021
Nanbukomugi	*Triticum aestivum* L.	Japan	*ppo-A1i*	*Ppo-D1a*	Figure 1
Yumekirari	*Triticum aestivum* L.	Japan	*ppo-A1i*	*Ppo-D1b*	Figure 1
Mexicali 75	*Triticum turgidum* ssp. durum	Italy	*ppo-A1i*	-	Nakamaru *et al.* 2023
KT120-012	*Ae. tauschii* L. var. typica	-	-	*ppo-D1d*	Figure S3
KT120-013	*Ae. tauschii* L. var. typica	-	-	*ppo-D1d*	Figure S3

Supplemental Table 2. Primer information.

forward primer	sequence 5'-3'	reverse primer	sequence 5'-3'	annealing temperature	reference or development
PPO16F	TGCTGACCGACCTTGACTCC	PPO16R	CTCGTCACCGTCACCCGTAT	66 -0.3 °C/cycle	He *et al.* 2007
PPO29F	TGAAGCTGCCGGTCATCTAC	PPO29R	AAGTTGCCCATGTCCTCGCC	66 -0.3 °C/cycle	He *et al.* 2007
STS01F	CGCCGACCATTTCAACA	STS01R	AGAAGGACCACAAGCCGTAG	57.5 °C	Wang *et al.* 2008
PPO18F	AACTGCTGGCTCTTCTTCCCA	PPO18R	AAGAAGTTGCCCATGTCCGC	66 -0.3 °C/cycle	Multiplex PCR
-	-	PPO18R-3	GGACACATCTCAAAGCAGAATCA	-	Nakamaru *et al.* 2023
ACTIN-F	GTTTCCTGGAATTGCTGATCGCAT	ACTIN-R	CATTATTTCATACAGCAGGCAAGC	65 °C	Sun *et al.* 2011
PPOD1seqF1	GCCAAGTTTCAACACGCTCC	PPOD1seqR1	CGAGAGCGGACCACATTAGG	50 °C	sequencing primer
SppoD1F-1	GCGGACCACATTAGGTAACTTCA	-	-	50 °C	sequencing primer
SppoD1F-2	GCCAGGCCAGTAGTACTAAT	-	-	50 °C	sequencing primer
SppoD1F-3	CGAGCAACACCGACTTCACC	-	-	50 °C	sequencing primer
SppoD1F-4	AACTTGTTGAAATGGTCGGCG	-	-	50 °C	sequencing primer
SppoD1F-5	TGGGAAGAAGAGCCAGCAGT	-	-	50 °C	sequencing primer
SppoD1F-6	GGCCTGGCGGTACATGATCT	-	-	50 °C	sequencing primer
RT-PPOD1-F	ATTGCATCCCTAACCCTAAGC	RT-PPOD1-R	GTCCGAAATCATCTGGCGGT	62 °C	RT-PCR primer
RT-PPOD1-F2	CAGCCTCCAAACCTTTCCGC	RT-PPOD1-R2	ATGAGAAGGACCACAAGCCG	62 °C	RT-PCR primer
		RT-PPOD1-R3	GCCACCAGTCCCATTAACTC	62 °C	
PPO-D1d_F1	TCCCAAGAGACCAGCAGATCG	PPO-D1d_R1	CGAACTTGACGAACTTGTTGAAATGG	60 °C	PCR primer
